# Novel Cu-MXene hybrid nanofluids for the experimental investigation of thermal performance in double pipe heat exchanger

**DOI:** 10.1038/s41598-025-94330-5

**Published:** 2025-03-22

**Authors:** Kodi Rajesh Kumar, Mohammed Rehaan Chandan, Bandaru Kiran, Aabid Hussain Shaik

**Affiliations:** Colloids and Polymers Research Group, School of Chemical Engineering, Vellore Institute of Technology, Vellore, Tamil Nadu 632014 India

**Keywords:** Hybrid nanofluids, Double pipe heat exchanger, Nusselt number, Heat transfer coefficient, Friction factor, Pressure drop, Log mean temperature difference, Energy science and technology, Engineering, Materials science, Nanoscience and technology

## Abstract

This work aims at analyzing the performance of a double-pipe heat exchanger (DPHE) using low concentrated (0.02–0.06 vol%) water, methanol, castor oil and silicon oil based Cu-MXene hybrid nanofluids. Convective heat transfer experiments were successfully performed in fabricated modular double pipe heat exchanger without using any twisted tapes, surfaces and effectively measured various heat transfer parameters such as Nusselt number (Nu), heat transfer coefficient (h), rate of heat transfer (Q), overall heat transfer coefficient (U), friction factor (*f*), pressure drop (∆P), and the thermal performance factor (TPF). The results indicated a considerable improvement in Nu, h, and U with some penalty in ∆P. Moreover, the TPF value of methanol and water-based hybrid nanofluids exceeded unity and showed superior heat transfer characteristics, confirming the viability of improving thermal performance in DPHE. Eventually, the LMTD and U of hybrid nanofluids were positively validated using Aspen HYSYS 12.1 version software to verify the experimental results and showed a variation of less than ± 5.35% for U and LMTD. These results successfully demonstrate the possible usage of proposed Cu-MXene hybrid nanofluids in thermal management systems, automotive cooling, and industrial cooling respectively. In addition, these findings also encourage its use in superior performance and cost-effective thermal management technologies.

**Supplementary Information:**

The online version contains supplementary material available at 10.1038/s41598-025-94330-5.

## Introduction

Nanofluids are initially developed by Choi and Eastman^1^ by dispersing nanosized particles in the range of < 100 nm into base fluids. These fluids show promising properties that may be tailored to specific demands. Several research groups have been motivated to investigate the unique properties of nanofluids over the past 3 decades to develop innovative thermal systems for a range of technological requirements. Increased thermal conductivity of nanofluids over traditional heat transfer fluids like water, oils, etc. is one of the unique advantages of mono nanofluids. These nanofluids are synthetically prepared using various types of nanoparticles such as metals, metal oxides (or) carbides of copper (Cu), silver (Ag), gold (Au), copper oxide (CuO), silicon dioxide (SiO_2_), aluminium oxide (Al_2_O_3_), titanium dioxide (TiO_2_), iron oxide (Fe_3_O_4_), single walled carbon nanotubes (SWCNT), multiwall carbon nanotubes (MWCNT), graphite (Gr), diamond, MXene etc^2–4^. Beyond mono nanofluids, researchers developed a completely new type of heat transfer fluid termed “Hybrid nanofluid” in order to further enhance the characteristics of mono nanofluids. Hybrid nanofluids are created by dispersing two or more different nanoparticles in base fluids^5–8^. Due to the presence of two types of different nanoparticles, these hybrid nanofluids have found widespread usage in biomedical^9^, automotive^10^, nuclear reactor^11^, heat pipe^12^, heat exchanger^13^, electronic industries^14^ and other sectors such as thermo-hydraulic performance enhancement applications^7^.

Generally, compact and energy efficient devices are usually chosen for enhancing the heat exchangers heat transfer performance^15,16^. It is essential to have heat exchangers to transfer heat across fluids of different temperatures. The efficiency with which working fluids convert heat is crucial in heat transfer applications. The efficacy depends on various thermophysical characteristics such as thermal conductivity, density, viscosity, and specific heat^17^. Various strategies were developed for enhancing heat exchanger performance and categorized into three distinct categories such as passive, active, and hybrid^18^. These classifications are distinguished in accordance with the utilization of an energy source. An external power source, such as a vibration or magnetic field, is essential for the active method. The passive method is superior and more widely used because it doesn’t require an external power source and also it only uses flow energy to improve heat transfer by employing fins, inserts, geometrical modifications etc^19–23^. A list of the recommendations given by different authors for improving heat exchanger performance is highlighted in Table 1.

**Table 1:** A comparative study representing the performance of heat exchangers.

Reference	Base fluid	Nanoparticles (NP)	Concentration (vol% and wt%)	Flow regime	HT performance techniques	Type of insert	Percentage enhancement (%)				
							Nu	h	ΔP	f	U
^15^	Water	Fe_3_O_4_ – SiO_2_	0.2–1	Laminar	Passive	–	25	21	23	21	–
^16^	Water	SiO_2_ – ZnO– CaO	0.01–0.05	Laminar	Passive	Twisted tape	107	–	25	43	–
^18^	Water	CuO	1	Laminar to turbulent	Active	–	–	66	–	21	277
^21^	Water	Al_2_O_3_	0.04–0.1	Laminar	Active	–	33	20	–	–	–
^24^	Water	Al_2_O_3_ –MWCNT	1.9	Turbulent	Passive	Chevron corrugated	10	20	5	8	15
^25^	Water	Fe_3_O_4_ –MWCNT	0–4	Turbulent	Passive	–	83	15	533	7	–
^26^	Water	Al_2_O_3_ –MWCNT	0–0.2	Laminar	Passive	Square cavity	6	43	–	–	–
^27^	Water	Al_2_O_3_ – CuO	0.6–1.8	Turbulent	Passive	twisted tape	43	11	–	31	–
^28^	Water	MWCNT –Fe_3_O_4_	0.1–0.7	Laminar	Passive	curved turbulator	2.27	16	–	–	–
^29^	Water	Al_2_O_3_ – TiO_2_	0.1	Laminar	Passive	–	16	16	–	–	–
^30^	EG	CoFe_2_O_4_ –BaTiO_3_	0–1	Laminar	Active	–	41	–	–	68	–
^31^	Water	TiO_2_	0.25–0.5	Laminar	Active	Fins	23	–	–	5	–

Moreover, some researchers also studied the behavior of heat exchangers using passive methods. Hussein^32^ synthesized EG based Aluminium nitride (AlN) nanofluids with concentrations ranging from 1 to 4% to investigate convective heat transfer in a double pipe heat exchanger under laminar flow conditions and observed 160% and 35% enhancement in heat transfer efficiency and thermal performance. Anitha et al.^33^ compared shell and tube heat exchanger performance using Cu and Al_2_O_3_ based mono and hybrid nanofluids ranging from 0.01 to 0.2 vol% concentrations and found that the hybrid nanofluids showed the maximum enhancement in heat transfer coefficient (h) ranging from75% − 139%, and also the efficiency of the HE was enhanced by 124% as compared to mono nanofluids. Irshad et al.^4^ experimentally examined the thermal performance of a tube heat exchanger under a turbulent flow regime using Therminol55 based hybrid nanofluids of MWCNT–CuO with concentrations ranging from 0.005 to 0.08 wt% and reported 128% enhancement in heat transfer coefficient followed by 38.4% enhancement in Nu and 103.88% enhancement in pumping power with a thermal performance factor of 1.31. Huminic and Huminic^34^ investigated four different water-based 0.1–0.3 vol% hybrid nanofluids synthesized using nanodiamonds (ND) with Nickel (Ni), Graphene oxide-Cobalt oxide (GO–Co_3_O_4_), MWCNT–Fe_3_O_4_, and ND–Fe_3_O_4_ under laminar and turbulent flow regime using figure of merit analysis and stated that hybrid nanofluids of ND–Ni and MWCNT–Fe_3_O_4_ are good candidates for heat transfer applications, while ND–Fe_3_O_4_ and GO–Co_3_O_4_ are not recommended due to reduced heat transfer coefficient and higher increment in pumping power.

However, there are few works conducted on simulation techniques to estimate hybrid nanofluids efficiency including stretched sheets^35^, rotating spheres^36^, and spongy voids^37^, using a magnetic field. Choudhary et al.^38^ numerically investigated heat transfer characteristics of TiO_2_-CuO/water hybrid nanofluid for a porous medium with a wedge shape and stated that enhancement in heat transfer was observed due to the transmission of wedge in the fluid’s direction. Likewise, Nasir et al.^39^ investigated the applicability of back-propagation artificial neural networks in conjunction with the Levenberg-Marquardt algorithm for evaluating heat transmission in hybrid nanofluids of EG-based MgO-GO under steady mixed convection flow over an exponentially stretched sheet. Similarly, Jat et al.^40^ studied flow features in a porous medium with non-Newtonian fluid under twisting action between twin concentric tubes. Nasir et al.^41^ estimated the efficiency of solar radiation by 3D radiative flow over a porous surface using artificial neural networks (ANN). In other works, Choudhary et al.^42^ investigated water-based Cu-Al_2_O_3_ hybrid nanofluids performance using numerical evaluation of nonlinear thermal radiation in the presence of microbes. They claim that hybrid nanofluids exhibited an improved Nusselt number compared to mono nanofluids and base fluid and additionally, stated that disk-shaped nanoparticles are more effective than spherical-shaped nanoparticles. Nasir et al.^43^ investigated EG-based tri-hybrid nanofluids of Cu-Al_2_O_3_-TiO_2_ by computational method to estimate heat sources, chemical reactions, radiation, and viscous dissipation under different slip conditions. Another arithmetical investigation by Jat et al.^44^ analyzed the repercussions of Dufour and Soret effects under a magnetic field on water-based CuO-Al_2_O_3_ hybrid nanofluid across an irregular flexible sheet in magnetohydrodynamics. In other studies, Choudary et al.^45^ conducted a numerical analysis to estimate the flow of a magnetohydrodynamic nanofluid by incorporating the movement of microorganisms in various arrangements such as flat plate, wedge, horizontal plate and concluded that Nu is more dominant for horizontal plate compared to standstill and wedge.

Moreover, other investigation studies estimate flow behavior and heat transmission of hybrid nanofluids by employing water-based SWCNT with MWCNT on a porous surface with the heat flux model of Cattaneo-Christov^46^. Nasir et al.^47^ studied the effect of water-based titanium oxide nanofluid and EG-based TiO_2_ nanofluid by employing the Darcy-Forchheimer equation in a radially stretched disk. Brownian motion and magneto-hydrodynamic migration of unstable 2D non-linear convective movement of a thin layer of nanofluid across an inclination stretched sheets were examined by Saeed et al.^48^. Gul et al.^49^ studied the effect of magnetic field on heat transfer using water-based hybrid nanofluids of Cu-Al_2_O_3_ by naturally occurring convection process within a permeable container and claim that the convective heat transmission inside the enclosure is improved as the Darcy and Rayleigh numbers improve. By taking into account of Gaussian Neural Network, Habib et al.^50^ investigated heat transfer in chemically reactive fluid flow considering Soret-Dufour effects using machine learning-driven analysis. Nasir and Berrouk^51^ stated that fluid motion improved due to a couple of stress parameters which are computed by Fick’s mass flux and Fourier’s energy. Alnahdia et al.^52^ investigated drug delivery systems and blood circulation by dispersing tri-hybrid nanoparticles of CuO-TiO_2_-Al_2_O_3_ in blood. Nasir et al.^53^ examined the consequences of thermal radiation and thermo-migration on the 3-D spinning nanofluid flow with SWCNT using Homotopy analysis (HAM).

In the current work, four various kinds of base fluids such as water, methanol, castor oil, and silicone oil are used to prepare hybrid nanofluids and improve the thermal performance of DPHE. Castor oil has been chosen as an effective base fluid in our study due to its high viscosity, lubrication, and good thermal stability which make it suitable for stabilizing the particles and also used for high-temperature applications. Moreover, silicon oil is considered a very effective coolant for transformer cooling applications. It also has high thermal stability, chemical inertness, hydrophobic nature, and a wide range of viscosities making it an effective base fluid especially in high temperature applications. These fluids are used as received from the supplier at room temperature. Hybrid nanoparticles were distributed at concentrations varying from 0.02 to 0.06 vol% to improve the thermophysical characteristics of base fluids. Studies on the specific characteristics of these hybrid nanofluids have previously been reported^54^. Most of the applications of these studies can be envisioned in sectors such as manufacturing, food and pharmaceuticals, power plants, generator cooling, etc.

In the current work, DPHE has been chosen as a heat transfer device due to its simple, straight forward design and low fabrication cost as compared to other types of HEX, such as shell and tube or plate type. In addition, a consistent flow pattern can be achieved, effective for low to medium heat transfer rates. Fouling, blockages or clogging is very low, and it can be easily configured for parallel or counter flow arrangemens.

From the overview of the literature, most of the research works were concentrated on enhancing the heat transfer process in heat exchangers by utilizing inserts, twists, fins, and high loading of nanoparticles. Also, it was noticed that hybrid nanofluids with metal and metal oxide nanoparticle combinations with high concentrations were used as coolants in heat exchangers. No work has been reported in enhancing the heat transfer performance in heat exchangers using a minimum concentration of metal and 2D layered structured hybrid material based nanofluids as coolants. In the current work, we proposed Cu-MXene hybrid nanofluids as an alternative to conventional coolants and conducted an experimental investigation of DPHE with very minimal volume concentrations of Cu and MXenes ranging from 0.02 to 0.06%. The objective of the present investigation is to explore the heat transfer metrics such as thermal performance factor, friction factor, Nusselt number, heat transfer coefficient, and pressure drop in double pipe heat exchanger under laminar flow conditions using Cu-MXene based hybrid nanofluid synthesized by dispersing very low concentration of hybrid nanoparticles (0-0.06 vol%) in four distinct base fluids i.e. methanol, water, silicone oil, and castor oil. In addition, this work also emphasized comparing the experimental heat transfer performance with the ASPEN HYSYS simulation software for verifying various heat transfer parameters.

## Experimental study

### Synthesis of Cu–MXene based hybrid nanofluids and associated thermophysical properties

**Fig. 1:**
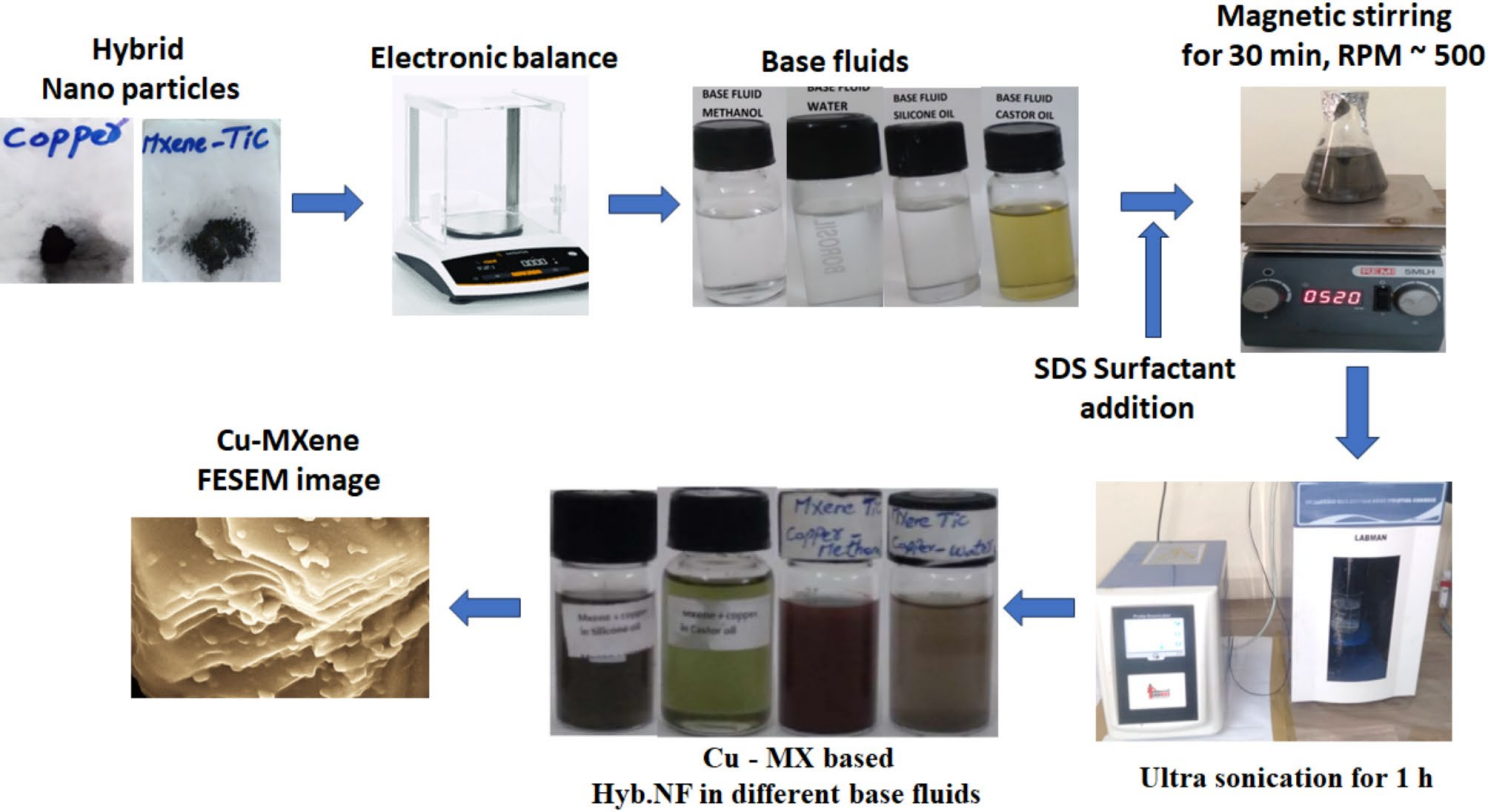
Synthesis of Cu-MXene hybrid nanofluid by two-step method.

Cu-MXene hybrid nanofluids are synthesized using two-step method by dispersing already synthesized MXene nanosheets and copper nanoparticles in base fluids as per the procedure reported in our previously published article^54^. As indicated in Fig. 1, pre-synthesized Cu nanoparticles at constant 0.01 vol% and MXene nanosheets with concentrations varying from 0.01 to 0.05 vol% are dispersed in different base fluids such as methanol, water, silicone oil, and castor oil containing sodium dodecyl sulfate (SDS) surfactant using magnetic stirring for 20 min at 500 rpm followed by ultra-probe sonication for an hour to prevent aggregation.

The essential parameters to be considered for evaluating heat transfer characteristics are density (ρ), Prandtl number (Pr), viscosity (µ), thermal conductivity (k), and specific heat capacity (Cp). These features were carefully investigated and collected from our previously published article^41^ and indicated in Tables 2, 3, 4 and 5.

**Table 2:** Methanol-based Cu-MXene hybrid nanofluids thermophysical parameters at 30 °C^54^.

Ф (vol%)	Cp (J/kg K)	µ (pa.s)	k (W/m. K)	Pr	ρ (kg/m^3^)
0	2560	0.000507	0.202	6.425347	778.1
0.02	2142.934	0.000580	0.232	5.35734	782.19
0.025	2095.174	0.000650	0.238	5.72211	784.72
0.03	2049.304	0.000730	0.261	5.73177	785.36
0.035	2005.217	0.000840	0.3	5.61461	785.55
0.05	1882.748	0.000930	0.335	5.22673	786.71
0.06	1808.444	0.001010	0.354	5.15968	786.80

**Table 3:** Water-based Cu-MXene hybrid nanofluids thermophysical parameters at 30 °C^54^.

Ф (vol%)	Cp (J/kg K)	µ (pa.s)	k (W/m. K)	Pr	ρ (kg/m^3^)
0	4178	0.0007972	0.598	5.572401	995.65
0.02	3695.64	0.00137	0.643	7.88556	997.43
0.025	3627.49	0.0014	0.653	7.7605	997.62
0.03	3561.37	0.00142	0.667	7.60328	997.90
0.035	3497.18	0.00144	0.685	7.35683	999.23
0.05	3315.41	0.00152	0.74	6.81451	999.52
0.06	3202.54	0.00165	0.887	5.93933	1001.99

**Table 4:** Silicone oil-based Cu-MXene hybrid nanofluids thermophysical parameters at 30 °C^54^.

Ф (vol%)	Cp (J/kg K)	µ (pa.s)	k (W/m. K)	Pr	ρ (kg/m^3^)
0	1200	0.2211	0.151	1757.086	946.48
0.02	1042.879	0.2408	0.154	1630.68	955.43
0.025	1025.031	0.3382	0.155	2236.55	955.62
0.03	1007.821	0.3883	0.158	2476.82	958.28
0.035	991.219	0.4117	0.161	2534.69	960.18
0.05	944.804	0.4298	0.165	2461.07	960.56
0.06	916.451	0.4399	0.17	2371.45	961.89

**Table 5:** Castor oil-based Cu-MXene hybrid nanofluids thermophysical parameters at 30 °C^54^.

Ф (vol%)	Cp (J/kg K)	µ (pa.s)	k (W/m. K)	Pr	ρ (kg/m^3^)
0	1900	0.34500	0.177	3703.39	939.38
0.02	1672.98	0.3537	0.204	2900.65	943.77
0.025	1642.29	0.3893	0.207	3088.62	946.87
0.03	1612.64	0.407	0.216	3038.62	951.11
0.035	1583.96	0.4143	0.223	2942.75	951.57
0.05	1503.38	0.465	0.243	2876.84	953.93
0.06	1453.83	0.493	0.261	2746.13	955.24

### Experimental setup

A modular simple double pipe heat exchanger is developed at the laboratory level by considering cost and Cu-MXene hybrid nanofluids for performing convective heat transfer studies. Table 6 represents the details of the exchanger consisting of extremely succinct and straight horizontal cylindrical pipes to enhance convective heat transfer performance without adopting inserts such as baffles or twisted tapes. In order to hold additional fluids, the exterior pipe is designed twice the size of the inner pipe.

**Fig. 2:**
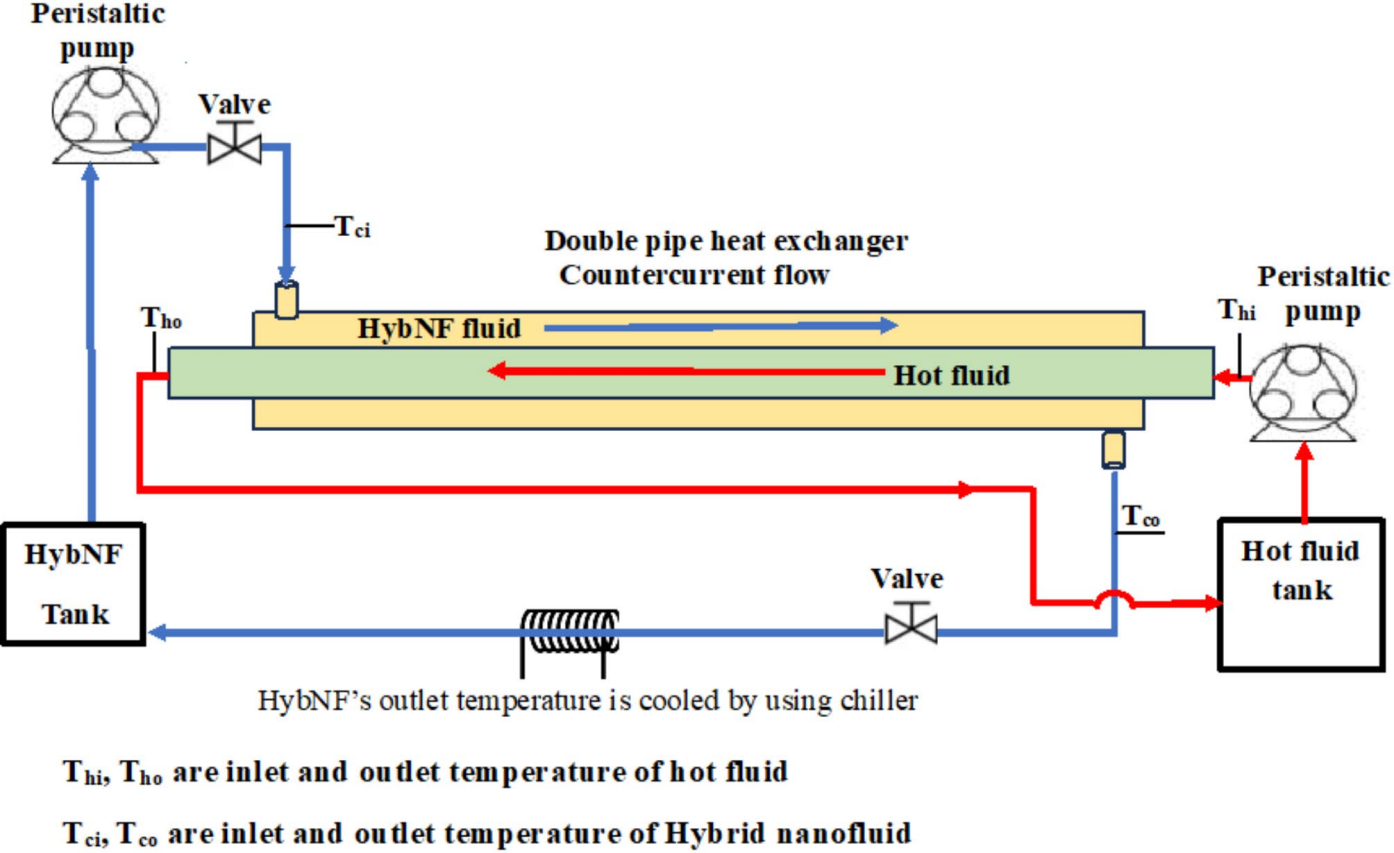
Schematic diagram showing DPHE.

The inlet temperatures of the hybrid nanofluids which acts as cold fluid and distilled water as hot stream were kept constant at 30°C and 50°C respectively and the output temperatures were measured using thermocouples. Peristaltic pumps are deployed to control the flow and chiller is used to maintain the cold fluid’s inlet temperature at 30°C as represented in Fig. 2. Since heat migrates from the hot fluid to the cold fluid, insulation was made using polyurethane foam (PU) with a thickness of 0.39” to eliminate the heat loss to the surroundings.

**Table 6:** Details regarding the pipe in pipe exchanger.

Parameters	Value
Length of the exchanger (L)	0.3 m
Material of construction	Stainless steel
Thermal conductivity of steel (k)	45 (W/m. K)
Shell inner diameter (D_i_)	0.008 m
Shell outer diameter (D_io_)	0.01 m
Tube inner diameter (D_o_)	0.016 m
Tube outer diameter (D_oo_)	0.021 m
Hot fluid inlet temperature (T_hi_)	323.15 K
Cold fluid inlet temperature (T_ci_)	303.15 K

The exchanger, comprising of four essential components like heating, cooling, and chilling, as well as a proportional-integral-derivative (PID) controller with indicator is outlined in Fig. 2. The hot fluid is contained in a 1” thick stainless-steel container to prevent heat loss to the surrounding environment, and it gets heated by an immersion heater. A PID controller is used to regulate the fluid’s temperature.

Firstly, pure base fluids like methanol, water, silicone oil, and castor oil were used in the experiment and circulated in the shell side of the heat exchanger for evaluating the heat exchanger’s performance at 30 °C. On the other hand, at an input temperature of 50 °C, the hot fluid stream was fed into the tube side at a Reynolds number 1220. Later, convective heat transfer studies have been carried out utilizing Cu–MXene hybrid nanofluids at volumetric concentrations from 0.02 to 0.06%, in the same conditions by replacing base fluids. Here, Cu-MXene nanofluids were circulated in the heat exchanger as a cold fluid and measured the temperatures at the inlet and outlet sections of the DPHE to analyze various convective heat transfer characteristics. A detailed discussion on experimental step-up was already reported in our previous work^55^.

### Data reduction

The hydraulic diameter of the inner tube (d_hi_) is the same as its diameter i.e., $\:{d}_{hi}={D}_{i}$, but for outer pipe (d_ho_) can be estimated mathematically using Eq. (1)^4^.

1 $$\:{d}_{ho}=\frac{4\:\times\:\:A}{P}=\:{D}_{o}{-\:D}_{i}$$

where A is the flow area (m^2^) and determined as ($\:\frac{\pi\:}{4}\:\times\:\left({D}_{o}^{2}-\:{D}_{i}^{2}\right))$, $\:{D}_{i}$ is inner tube diameter, $\:{D}_{o}$ is outer shell diameter, and P is wetted perimeter.

The Prandtl number (Pr) of the hybrid nanofluids’ are figured out by using Eq. (2) with the help of viscosity, specific heat, and thermal conductivity respectively^4^.

2 $$\:{Pr}_{hnf}=\:{\left(\frac{{\upmu\:}\:\times\:Cp}{k}\right)}_{hnf}$$

The heat transfer rates (Q) for hold and cold fluids, reveilles the heat gain of the cold fluid (Q_hybnf_) and the heat loss of the hot fluid (Q_h_) should be equal with reference to the energy balance equation i.e. Q_hybnf_ = Q_hf_, from the current studies, an average of 4.21% deviation is observed, the rate of heat flow in tube side and annulus side are computed utilizing Eq. (3a) and (3b)^4^.

3a $$Q_{{hybnf}} = ~Cp_{{hybnf}} \times \dot{m}_{{hybnf~}} \times (T_{{hybnf,o}} - T_{{hybnf,i}} )$$

3b $$Q_{{hf}} = \dot{m}_{{h~}} \times Cp_{h} \times (T_{{h,i}} - ~T_{{h,o}} )$$

Equation (3c) provides the average rate of heat transfer in watts

3c $$\:{Q}_{avg}=\:\frac{{(Q}_{hf}+\:{Q}_{hybnf})}{2}$$

where Q is Heat transfer rate, subscripts i and o stands for inlet and outlet respectively, ṁ mass flow rate of fluid, $\:{T}_{hybnf}$ temperature of cold fluid, Cp specific heat, and $\:{T}_{hf}$ temperature of hot fluid respectively.

“Re” is used to indicate type of flow regime based on the fluid’s flow rates, density, diameter, and viscosity. By utilizing Eq. (4a and 4b) “Re” is estimated for hot stream as well as for cold stream^16^.

4a $$\text{Re} _{{hf}} = \left( {\frac{{\rho ~ \times ~D_{i} ~ \times ~v}}{\mu }} \right)$$

4b $$\text{Re} _{{hybnf}} = \left( {\frac{{v \times ~D_{o} \times ~\rho }}{\mu }} \right)$$

where$\:{{Re}_{hf}\:and\:Re}_{hybnf}$denotesRe for hot fluid and hybrid nanofluids respectively.

For the counterflow pattern, the logarithmic mean temperature difference (LMTD) is computed from Eq. (5) below^15^.

5 $$\:{\varDelta\:T}_{LMTD}=\:\frac{\left({T}_{hf,i}-\:{T}_{c,o}\right)-\left({T}_{hf,o}-\:{T}_{c,i}\right)}{ln\frac{\left({T}_{hf,i}\:-\:{T}_{c,o}\right)}{\left({T}_{hf,o}-\:{T}_{c,i}\right)}}$$

where $\:{T}_{hf,i}\:{and\:T}_{hf,o}$ are temperatures of hot fluid at inlet and outlet, $\:{T}_{c,i}\:and\:{T}_{c,o}$ are temperature of cold fluid at inlet and outlet respectively.

Equation (6) is used to calculate the average temperatures of hot fluid ($\:{T}_{hf\:avg})$ and cold fluid ($\:{T}_{c\:avg})$

6 $$\:{T}_{hf\:avg}=\:\frac{{(T}_{h,o}+\:{T}_{h,i})}{2}{and\:T}_{c\:avg}=\:\frac{{(T}_{c,o}+\:{T}_{c,i})}{2}$$

The overall heat transfer coefficient (U) is computed by using Eq. (7)^18^,

7 $$\:U=\left(\frac{{Q}_{avg}}{LMTD\:\times\:\:A}\right)$$

where Q_avg_ average heat transfer rate (W), U is overall heat transfer coefficient (W/m^2^. K), and A area of heat transfer (m^2^).

The Sieder and Tate equation has been used to calculate the Nusselt number since the determined Reynolds number (Re) < 2300, which is classified as laminar flow^56,57^. The following Nusselt number Eq. (8a) and (8b) were applied to the shell side and tube side of DPHE respectively.

8a $$\:{Nu}_{o}=1.86\times\:{\left(\frac{{{P}_{r\:}\times\:\:{D}_{o}\times\:\:Re}_{nf}}{L}\right)}^{0.333}\times\:{\left(\frac{{\mu\:}_{b}}{{\mu\:}_{w}}\right)}^{0.14}$$

8b $$\:{Nu}_{i}=1.86\times\:{\left(\frac{{{P}_{r\:}\times\:\:{D}_{i}\:\times\:\:Re}_{i}}{L}\right)}^{0.333}\times\:{\left(\frac{{\mu\:}_{nf}}{{\mu\:}_{w}}\right)}^{0.14}$$

$\:{\:\mu\:}_{w}$is determined using the wall temperature and T_wall_ could be approximated from Eq. (9)^37,38^:

9 $$\:{T}_{wall}\cong\:\:0.5\times\:\left(\left(\frac{{T}_{i}+{T}_{o}}{2}\right)+\left(\frac{{t}_{o}+{t}_{i}}{2}\right)\right)$$

The hydraulic diameter (d_ho_), the Nusselt number (Nu), and the thermal conductivity values of hybrid nanofluids (k_hybnf_) have been applied for estimating the annulus heat transfer coefficient (h_o_), by using Eqs. (10) and (8a) respectively^18^.

10 $$\:{h}_{o}=\:\frac{{(N}_{uo}\times\:{k}_{hybnf})}{{d}_{ho}}$$

Equation (11) represents friction factor under laminar flow in tube side as well as shell side and computed by using the Darcy friction factor equation. For, flow through annulus i.e., outer pipe (D_o_), equation is given as^57^:

11a $$\:{f}_{i}={\frac{64}{Re}}_{i}$$

11b $$\:{f}_{o}={\frac{64}{Re}}_{nf}\times\:{\left(\frac{{(1+k}^{2})}{{\left(1-k\right)}^{2}}+\:\frac{\left(1+k\right)}{\left(1-k\right)\times\:\:lnk}\right)}^{-1}$$

where k = $$\:\frac{{D}_{i}}{{D}_{o}}$$

Equation (12) has been utilized to derive the pressure drop (∆P) in a heat exchanger

12a $$f_{{hf}} = \frac{{\Delta P_{{hf}} }}{{\left( {\frac{L}{{D_{i} }}} \right)~ \times ~\left( {\frac{{v^{2} ~ \times ~\rho _{{hf}} }}{2}} \right)}}$$

12b $$f_{{hybnf}} = \frac{{\Delta P_{{hnf}} }}{{\left( {\frac{L}{{D_{o} }}} \right)~ \times ~\left( {\frac{{v^{2} \times ~\rho _{{hybnf}} }}{2}} \right)}}$$

where $\:v$ is velocity of the fluid in (m/s)

The following Eq. (13) was used to evaluate the performance evaluation criteria (PEC) (or) thermal performance factor (TPF)defined by Webb et al.^58^ by considering Nusselt number enhancement ratio to frictional losses^13,19,25,58–60^.

13 $$\:PEC=\frac{\left(\frac{{Nu}_{hybnf}}{{Nu}_{bf}}\right)}{{\left(\frac{{f}_{hybnf}}{{f}_{bf}}\right)}^{\left(\frac{1}{3}\right)}}$$

where $\:{Nu}_{hybnf}$, $\:{Nu}_{bf}$, $\:{f}_{hybnf}$, and $\:{f}_{bf}$ are Nusselt number and frictional losses for hybrid nanofluids and base fluids respectively.

Equation 14 is used to obtain percentage deviation while evaluating accuracy in terms of deviation^61^.

14 $$\:\%\:Deviation=\frac{\left(Predicted\:-\:Experimental\right)}{Experimental}\times\:100$$

The following Eq. (15) was applied to figure out the percentage enhancement.

15 $$\:\%\:e=\left(\frac{{P}_{hybnf}-\:{P}_{bf}}{{P}_{bf}}\right)\times\:100$$

where P stands for attributes such as the LMTD, Nu, h, *f*, ΔP, and U respectively. While “$\:e"$ stands for the percentage of enhancement.

### Limitations

There are some limitations of the current proposed work and these are listed below.

- As the hybrid nanofluid were synthesized using low concentrations of nanoparticles, agglomeration and saturation effects at high concentrations need to be explored.
- Measurement of heat transfer characteristics under turbulent flow conditions were not considered as fabricated equipment is designed for laminar conditions.
- In addition, long-term operational conditions associated with clogging, fouling, and degradations of hybrid nanofluids were not fully explored.
- Scaling up to a large scale is also another limitation that need to be considered further.

## Results and discussions

The main objective of the proposed work is to enhance the convective heat transfer performance of fabricated modular double-pipe heat exchanger (without any inserts/baffles) using very low concentrated Cu-MXene hybrid nanofluids. In order to accomplish the maximum enhancement, various heat transfer properties such as heat transfer rate, LMTD, heat transfer coefficients, Nusselt number, friction factor, pressure drop, etc. need to be analyzed with respect to the flow rate and concentration of hybrid nanoparticles. A detailed analysis representing the effect of all these heat transfer parameters is discussed in the subsequent sections.

### Effect of Reynolds number and concentration of Cu-MXene nanoparticles on heat transfer rate (Q)

**Fig. 3:**
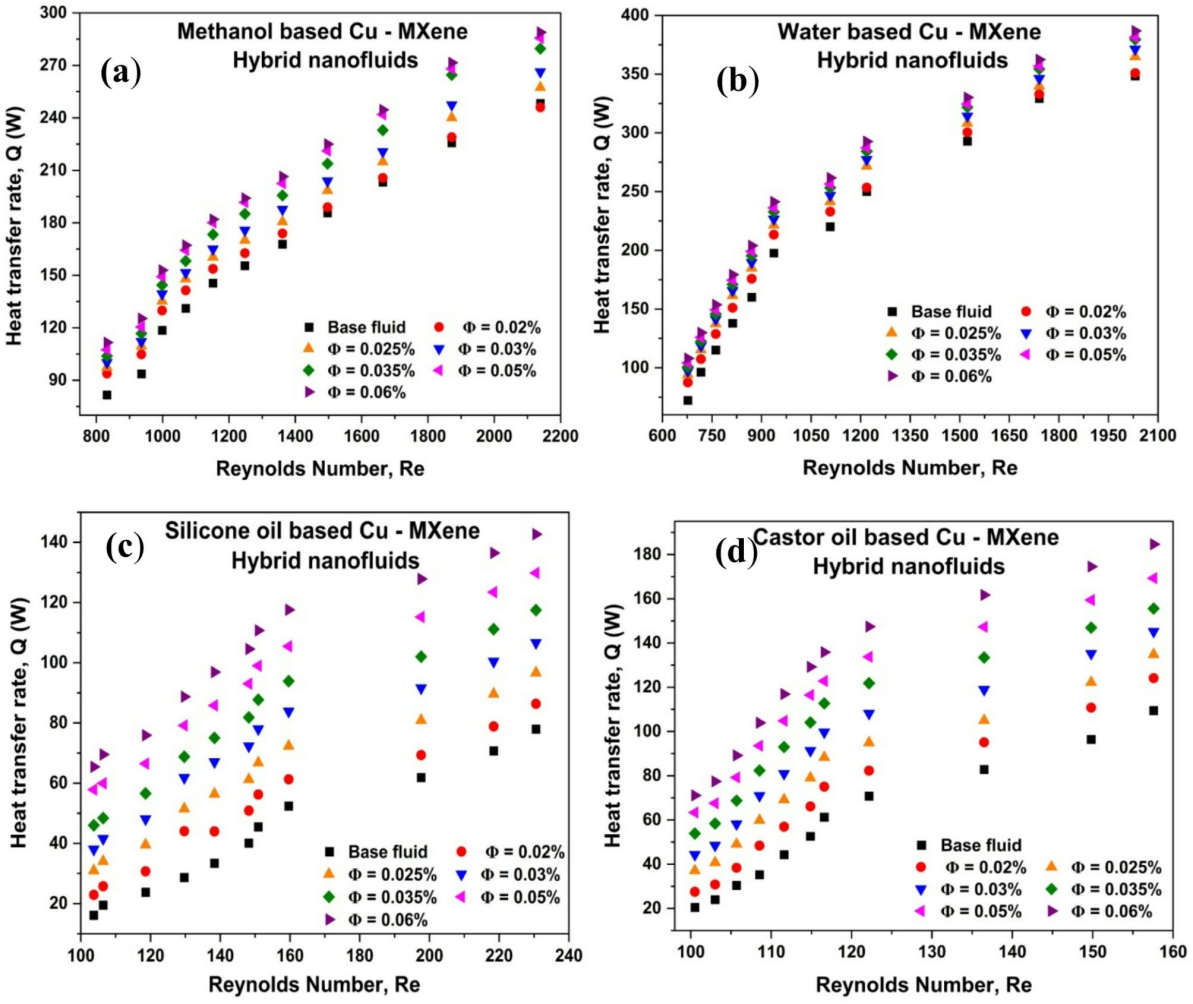
Effect of Cu–MXene concentration and Reynolds number on heat transfer rate for (**a**) methanol-based, (**b**) water-based, (**c**) silicone oil-based, and (**d**) castor oil based.

The hybrid nanofluids acting as cold fluid and distilled water representing as hot fluid were passed into shell side and tube side at a constant inlet temperature of 303.15 K and 323.15 K respectively using peristaltic pumps. The flow of hot fluid is fixed at 1220 Re for entire experiments and cold stream is varied step by step. Effect of Re and concentration of hybrid nanoparticles on heat transfer rate is shown in Fig. 3. From Fig. 3a–d, it has been observed that the rate of heat transfer rate (Q) enhances with an increase in Re and concentration of hybrid nanoparticles possibly due to increase in thermal conductivity and the flow rate. The heat transfer rates are improved by 25.28%, 24.29%, 172%, and 149.34% for methanol-based, water-based, silicon oil-based, and castor oil-based Cu-MXene nanofluids prepared using concentrations of 0.01% (Cu) and 0.05% (MXene). From Fig. 3, Q is increasing concerning Re and concentration for all hybrid nanofluids. This means that heat transfer is better, which makes them perfect for large-scale industrial uses.

### Effect of Reynolds number and concentration of hybrid nanoparticles on log mean temperature difference (LMTD) and validation using ASPEN HYSYS software

**Fig. 4:**
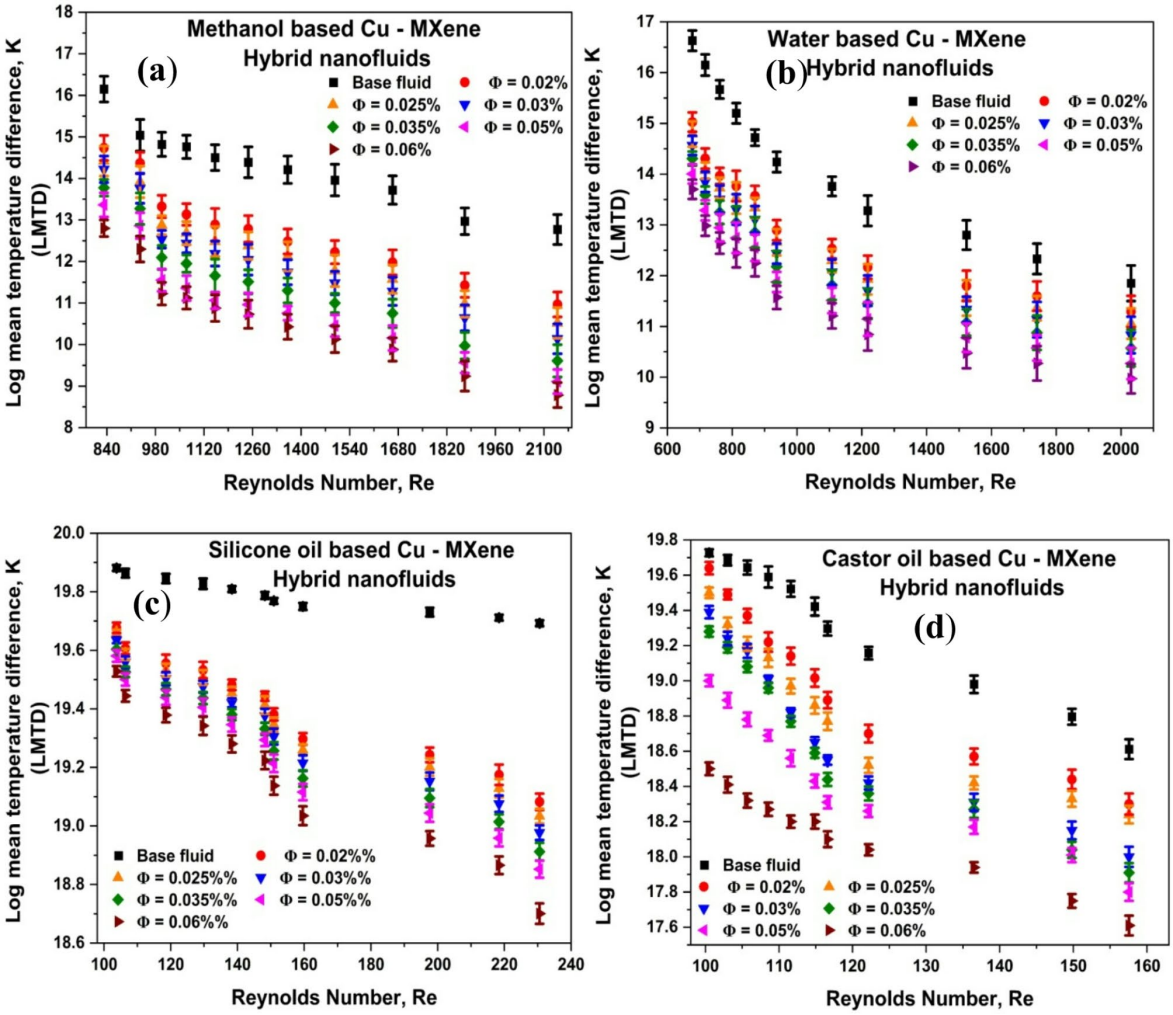
Effect of Cu–MXene concentration and Reynolds number on LMTD for (**a**) methanol-based, (**b**) water-based, (**c**) silicone oil-based, and (**d**) castor oil-based hybrid nanofluids.

Heat exchanger’s thermal performance is used to assess the temperature difference between the hot and cold streams and it can be measured by LMTD using Eq. (5). The influence of concentration of Cu-MXene hybrid nanofluids and Re on LMTD was represented in Fig. 4a–d. From the Fig. 4, the LMTD drops as the concentration of Cu-MXene increases suggesting a wider dispersion of thermal capacity^62^. A substantial percentage drop in LMTD values can be observed from Table 7 as compared to base fluids by utilizing hybrid nanofluids. Heat exchanger efficiency can be improved, and the size of the exchanger can be reduced.

Furthermore, LMTD is validated by the NRTL thermodynamic fluid package approach in the Aspen HYSYS simulation 12.1 version. Fig. S1 and S2 in the supplemental material displays a depiction of the HYSYS simulator and the validated LMTD values for methanol-based, water-based, silicone oil-based, and castor oil-based hybrid nanofluids were reported in the supplementary information as Fig. S3. The average percentage deviation is predicted from Eq. 14 for Cu-MXene hybrid nanofluids and found approximately − 0.3%, 0.75%, -1.52%, and − 0.29%for methanol-based, water-based, silicone oil-based, and castor oil-based respectively.

**Table 7:** Table indicating the corresponding reduction in LMTD values relative to base fluids.

Ф (vol%)	Mean LMTD percentage drop for various hybrid nanofluids			
	Methanol-based Cu - MXene HYBNF	Water-based Cu - MXene HYBNF	Silicone oil-based Cu - MXene HYBNF	Castor oil-based Cu - MXene HYBNF
0.02	10.90	8.58	1.93	1.77
0.025	13.84	10.26	2.09	2.47
0.03	15.93	11.86	2.29	3.21
0.035	19.51	13.69	2.52	3.60
0.05	23.15	15.83	2.72	4.53
0.06	25.48	17.98	3.12	6.19

### Effect of Reynolds number and concentration of hybrid nanoparticles on overall heat transfer coefficient (U)

**Fig. 5:**
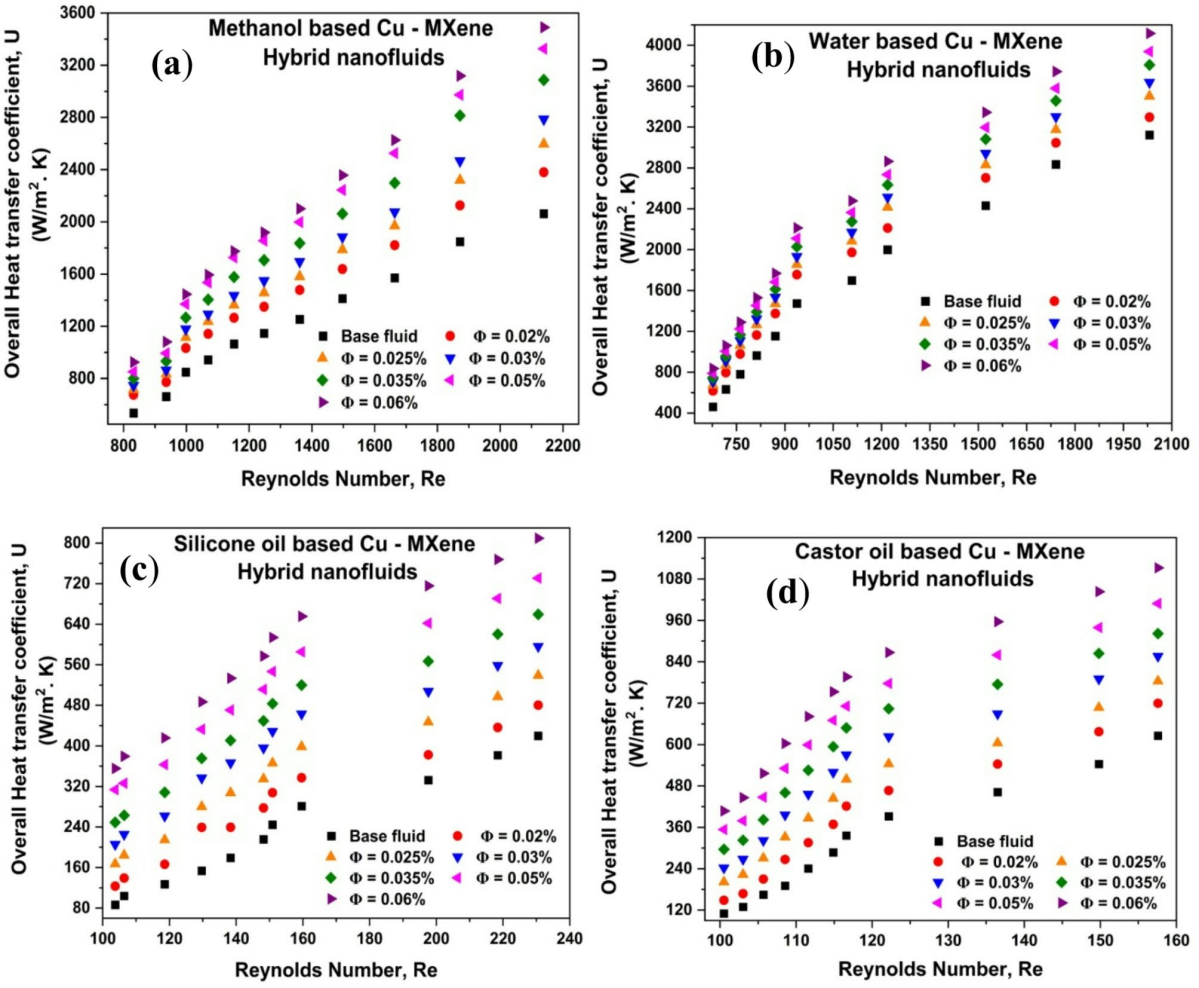
Effect of Cu-MXene concentration and Reynolds number on overall heat transfer coefficient for (**a**) methanol-based, (**b**) water-based, (**c**) silicone oil-based, and (**d**) castor oil-based hybrid nanofluids.

The overall heat transfer coefficient for all the hybrid nanofluids is calculated using Eq. 7 and shown in Fig. 5. From Fig. 5a–d, it can be seen that the overall heat transfer coefficient increases with an increase in nanoparticle concentration for all the Cu-MXene hybrid nanofluids due to the decrease in the LMTD. The rise in U may also be vindicated by the decline in thermal resistance and enhancement of heat coefficient of hybrid nanofluids as reported by other researchers^15^. The percentage enhancement of U as compared to base fluids with an increase in the concentration of hybrid nanoparticles is calculated and shown in Table 8. Additionally, Aspen HYSYS 12.1 software was used to validate the results, which were presented as supplementary information in Fig. S4. The percentage deviation for the methanol-based, water-based, silicone oil-based, and castor oil-based hybrid nanofluids was estimated using Eq. (14) and reported as-5.35%, -1.24%, 0.05%, and 1.57% respectively. From Fig. 5, it can also be inferred that these fluids are beneficial for power plants, heating, ventilation, and air conditioning (HVAC) either for heating or cooling purposes due to the enhancement of U over Re.

**Table 8:** Enhancement percentage of U compared to their respective base fluids.

Ф (vol%)	U compared to their respective base fluids in terms of % enhancement			
	Methanol-based Cu-MXene Hyb. NF	Water-based Cu-MXene Hyb. NF	Silicone oil-based Cu-MXene Hyb. NF	Castor oil-based Cu-MXene Hyb. NF
0.02	18.49	17.84	20.01	25.79
0.025	27.97	26.30	47.02	52.07
0.03	35.09	31.73	71.70	76.14
0.035	47.89	38.34	99.21	102.03
0.05	59.93	44.31	122.81	127.33
0.06	68.14	51.64	153.35	156.68

### Effect of Reynolds number and concentration of Cu-MXene nanoparticles on Nusselt number

**Fig. 6:**
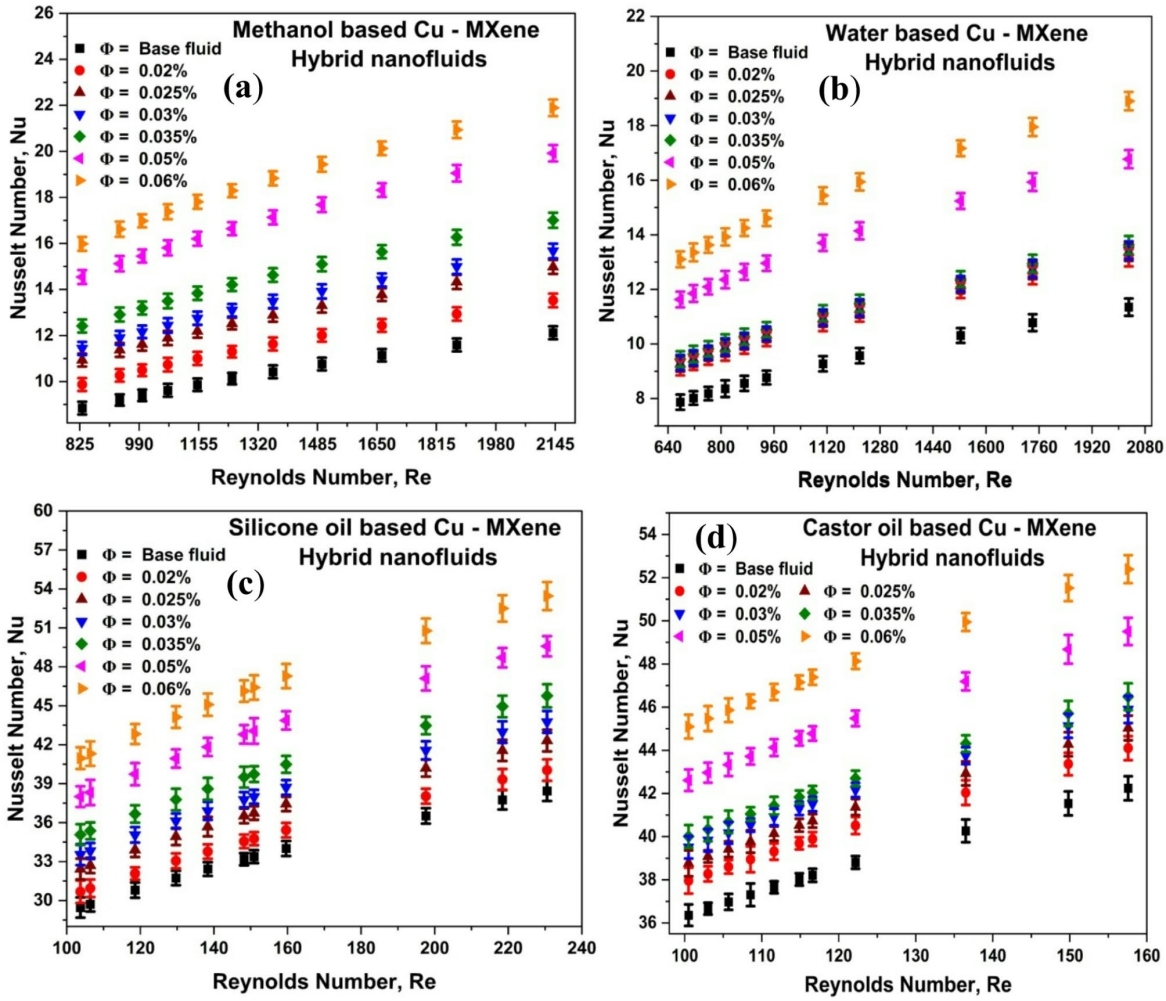
Effect of Cu-MXene concentration and Reynolds number on overall Nusselt number for (**a**) methanol-based, (**b**) water-based, (**c**) silicone oil-based, and (**d**) castor oil-based hybrid nanofluids.

Nusselt number is estimated using Eq. (8) for all the hybrid nanofluids with respect to the concentration of nanoparticles and shown in Fig. 6. From Fig. 6, it has been noticed that the Nusselt number increases with the increase in the Re and concentration of nanoparticles. This enhancement could be possibly due to the increased turbulence that occurs as the Re increases and better thermophysical properties due to dispersion of Cu-MXene nanoparticles in the base fluids as also reported by other researchers^63,64^. The maximum enhancement of Nu for methanol-based, water-based, silicone oil-based, and castor oil-based hybrid nanofluids are found to be80.6%, 66%, 42.9%, and 24%, respectively, at 0.06 volume concentration of hybrid nanoparticles. Hence, these hybrid nanofluids can be beneficial for refrigeration, heat recovery systems, and cooling of electronics due to is high values of Nu.

Convective heat transfer experiments were conducted using synthesized Cu-MXene hybrid nanofluids to validate the Nusselt number under laminar flow conditions. The Shah correlation and Hausen correlation^65,66^ represented in Eqs. (16,17) revealed a satisfactory level of agreement with the experimental Nusselt number as shown in the supplementary information (Fig. S5 and S6). Hybrid nanofluids prepared using 0.02 vol% concentration fits better as compared to other concentrations with R^2^ as 0.972–0.993 as shown in Fig. S6.

If $$\:{{Re}_{hynf}\times\:Pr}_{hybnf}\times\:\left(\frac{{D}_{o}}{L}\right)\ge\:33.33,\:\:\text{t}\text{h}\text{e}\text{n}$$

16 $$\:{Nu}_{hybnf}=1.953\times\:\left({\left(\left(\frac{{D}_{o}}{L}\right)\times\:Pr{\times\:Re}_{hybnf}\right)}^{0.3333}\right)$$

If$$\:0.1<{Pr}_{hybnf\:}\times\:\left(\frac{{d}_{e}}{L}\right)\times\:{Re}_{hybnf}<{10}^{4}$$, then

17 $$\:{Nu}_{hybnf}=3.66+\left(\frac{\left(0.19\:\times\:{\left({{Re}_{hybnf}\times\:Pr}_{hybnf\:}\times\:\left(\frac{{D}_{o}}{L}\right)\right)}^{0.8}\right)}{\left(1+0.117\:\times\:{\left({\left(\frac{{D}_{o}}{L}\right)\times\:Pr}_{hybnf\:}\times\:{Re}_{hybnf}\right)}^{0.467}\right)}\right)$$

where $\:{Nu}_{hybnf}$ represents hybrid nanofluids Nusselt number, $\:{Pr}_{hybnf\:}$shows hybrid nanofluids Prandtl number, $\:{Re}_{hybnf}$ signifies hybrid nanofluids Reynolds number, $\:{D}_{o}$designates outer pipe diameter, and L represents length of heat exchanger.

### Effect of Reynolds number and concentration of Cu-MXene nanoparticles on the heat transfer coefficient (h)

**Fig. 7:**
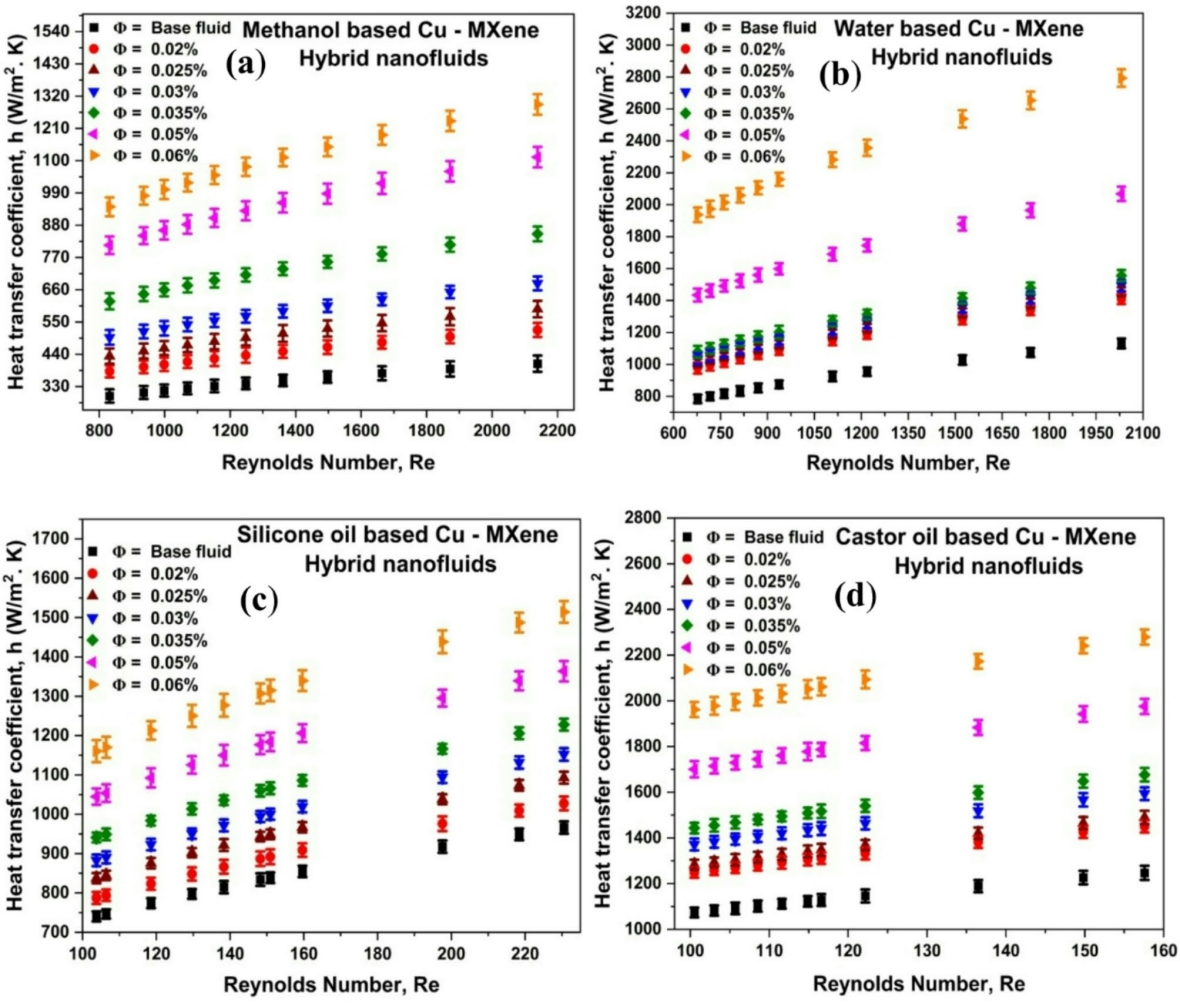
Effect of Cu-MXene volume concentration and Reynolds number on Heat transfer coefficient for (**a**) methanol-based, (**b**) water-based, (**c**) silicone oil-based and, (**d**) castor oil-based hybrid nanofluids.

Equation (10) is used to compute the hybrid nanofluid heat transfer coefficient (h_hybnf_) with respect to various concentrations of hybrid nanoparticles. Figure 7 displays the effect of Re and concentration of hybrid nanoparticles on the heat transfer coefficient (h). From the Fig. 7, it has been witnessed that the heat transfer coefficient of all the hybrid nanofluids improved as the Re elevated due to increase in turbulence and heat transfer rate. In comparison to lesser turbulence at lower Re, hybrid nanofluids with higher turbulence at higher Re absorb more heat and exhibit a higher heat transfer rate. As a result, for all hybrid nanofluids, the heat transfer coefficient elevates at high Re and diminishes at low Reynolds numbers. This kind of phenomenon was also reported by other researchers^29,63^. Moreover, increase in thermal conductivity with increase in concentration of hybrid nanoparticles and Brownian motion of particles by impact and interaction due to change in flow rates of fluids also helps in enhancing the^20^. The heat transfer coefficients improved by 216%, 146%, 60%, and 82% at volume concentrations of 0.06% for methanol-based, water-based, silicone oil-based, and castor oil-based solutions, respectively. Hence silicone oil-based hybrid nanofluids can be utilized for lubrication, castor oil-based can be used for equipment cooling, and the rest of the fluids can be used for other heat transfer applications.

### Effect of Reynolds number and concentration of hybrid nanoparticles on friction factor (f)

**Fig. 8:**
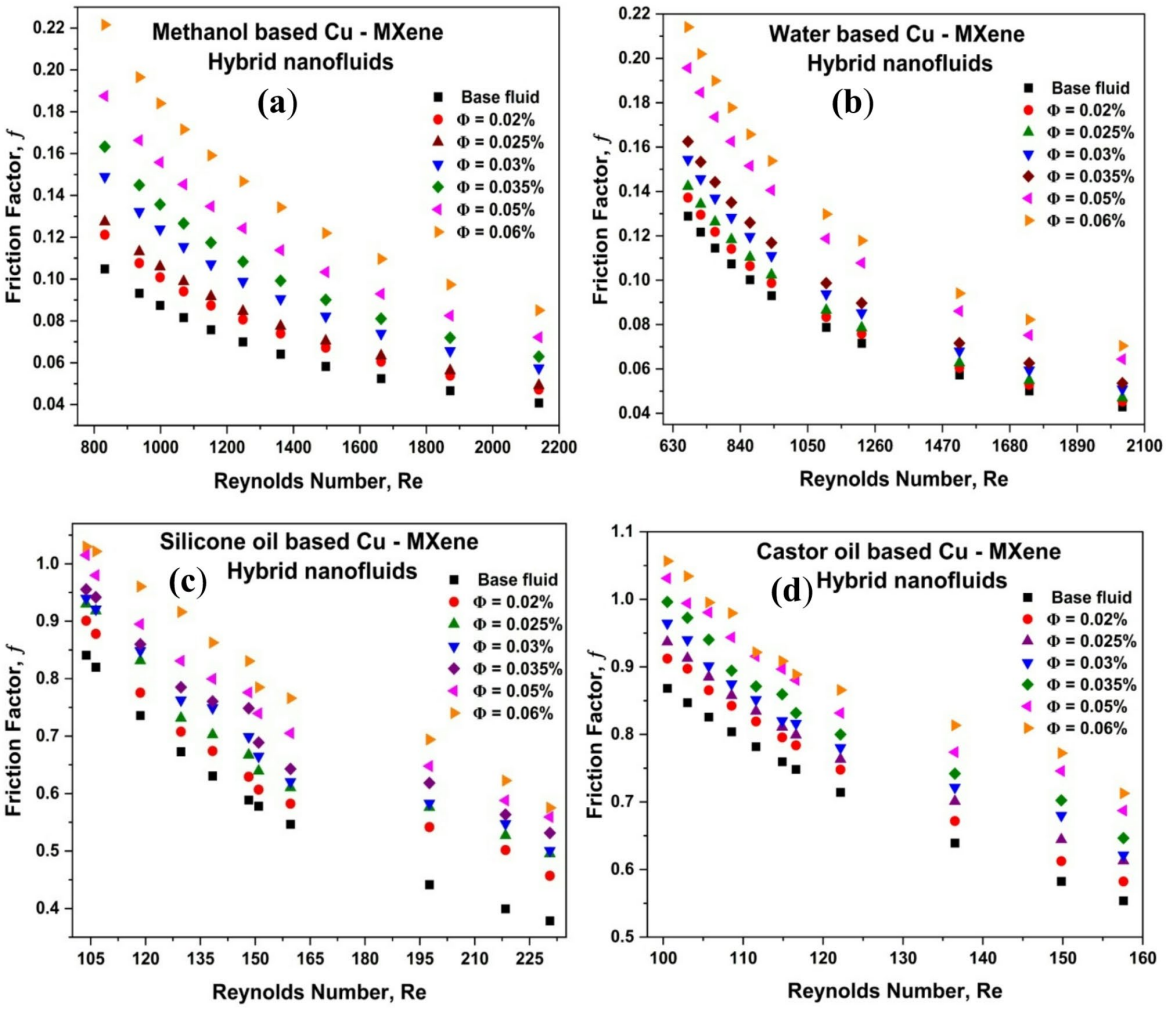
Effect of Cu-MXene volume concentration and Reynolds number on friction factor for (**a**) methanol-based, (**b**) water-based, (**c**) silicone oil-based, and, (**d**) castor oil-based hybrid nanofluids.

Friction factor plays a major role in various commercial and practical applications. The effect of Re and concentration of hybrid nanoparticles on friction factor is shown in Fig. 8. From the Fig. 8, it has been detected that the friction factor declines with increasing Re but amplifies with increasing concentration of hybrid nanoparticles. Friction factor of hybrid nanofluids is higher than that of base fluids due to an improvement in hybrid nanofluids’ Brownian motion and a significant enhancement in thermophysical characteristics^5,23^. Furthermore, the rises in viscosity and additional shear stress that are applied to the pipe wall generate a stronger friction coefficient. The percentage enhancement of friction factor of the methanol-based, water-based, silicone oil-based, and castor oil-based Cu-MXene hybrid nanofluids are reported as108%, 63%, 52%, and 28%, respectively at 0.06 vol% concentration of hybrid nanoparticles.

### Effect of Reynolds number and concentration of hybrid nanoparticles on pressure drop

**Fig. 9:**
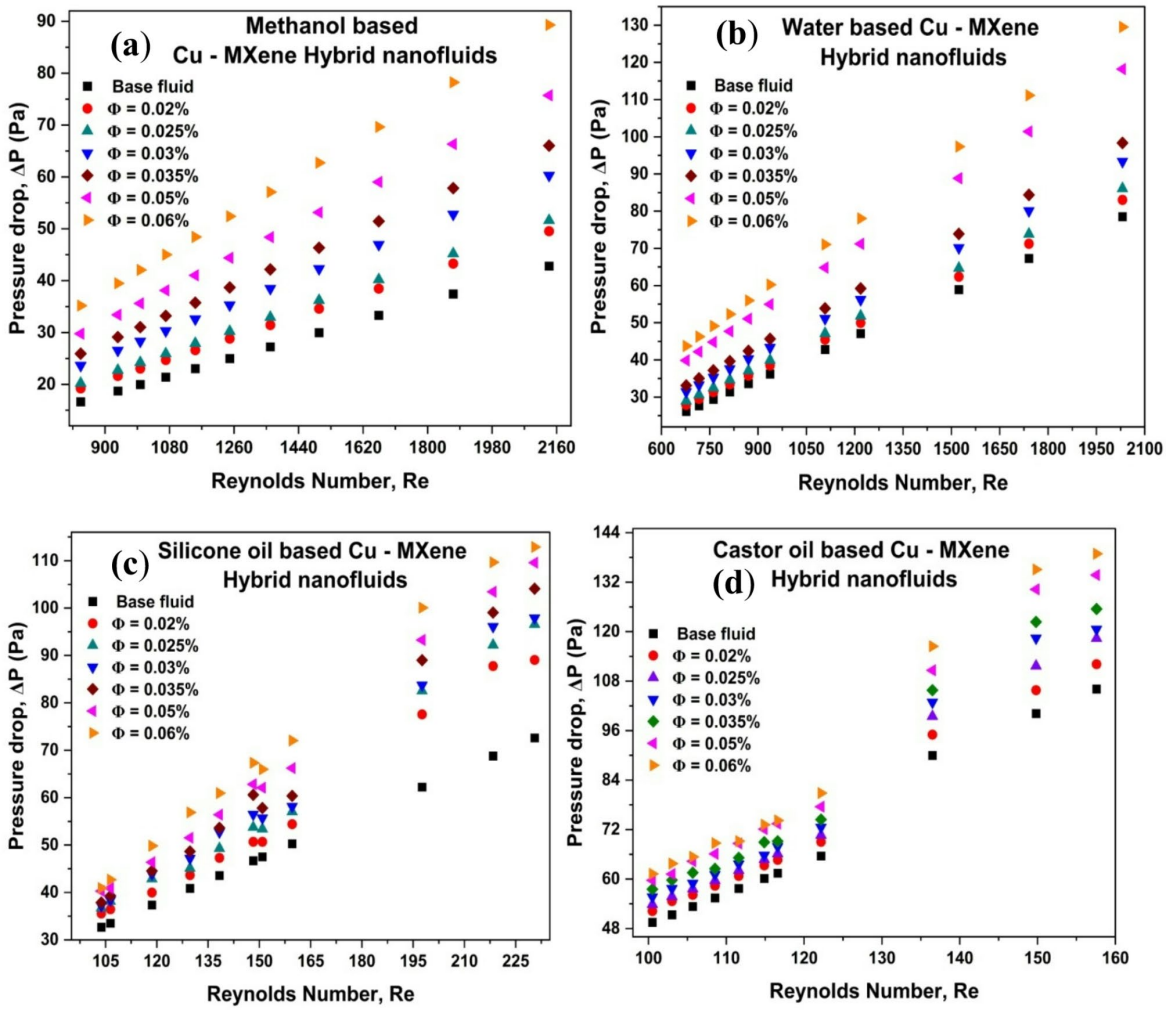
Effect of Cu-MXene volume concentration and Reynolds number on pressure drop for (**a**) methanol-based, (**b**) water-based, (**c**) silicone oil-based and, (**d**) castor oil-based hybrid nanofluids.

The practical application of heat exchangers depends on the fluid’s heat-transfer efficiency and pressure drop characteristics. Nanoparticles add little to no alteration in the properties of any base fluid; however, they significantly alter the pressure drop^15,66^. Figure 9 shows the effect of Re and concentration of hybrid nanoparticles on the pressure drop. From the Fig. 9, it can be seen that the pressure drop of Cu-MXene based hybrid nanofluids is greater than that of the base fluids and increase with increase in Re and concentration of hybrid nanoparticles. Higher viscosity of hybrid nanofluids in comparison to base fluids leads to increased flow resistance, and as nanofluids becomes denser and more viscous, high concentrations of hybrid nanofluids instantly lead to an increase in pressure drop^15^. Cu-MXene based hybrid nanofluids exhibited pressure drop enhancement of 113%, 65%, 56%, and 31% for methanol-based, water-based, silicone oil-based, and castor oil-based, respectively (Fig. 9a–d). The existence of high concentrations, fluid flow rate^63^, and thermophysical characteristics^26^ are responsible for the enhancement in pressure drop. It can be inferred that methanol-based hybrid nanofluids can be applicable for low viscosity cooling equipment’s and water-based hybrid nanofluids can be suitable for heat exchangers. Whereas silicone oil-based and castor oil-based hybrid nanofluids are more suitable for high temperature lubrication.

### Effect of Reynolds number and concentration of hybrid nanoparticles thermal performance factor (TPF)

**Fig. 10:**
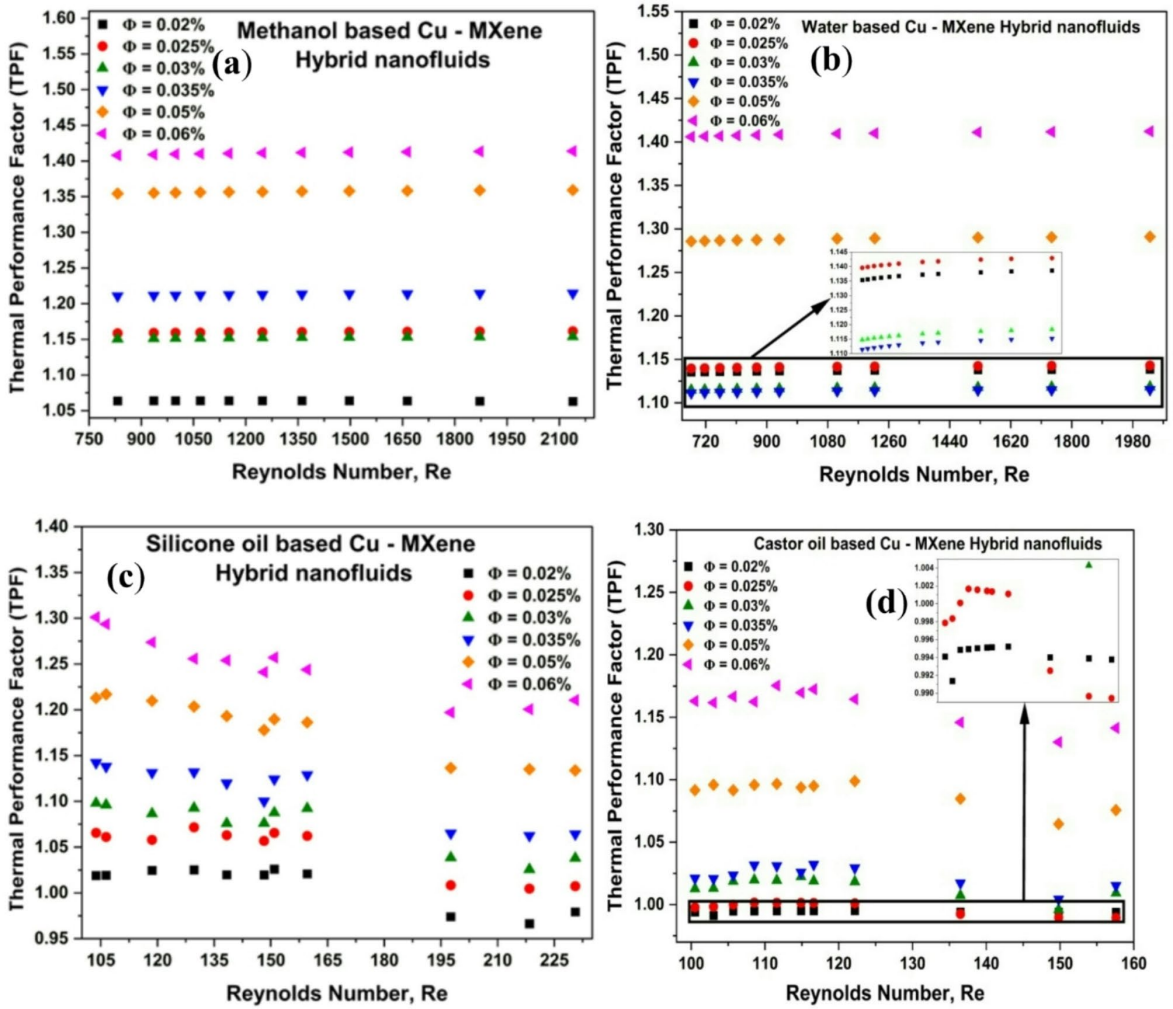
Effect of Cu-MXene volume concentration and Reynolds number on thermal performance factor (TPF) for (**a**) methanol-based, (**b**) water-based, (**c**) silicone oil-based and, (**d**) castor oil-based hybrid nanofluids.

In addition to above mentioned heat transferred parameters and pressure drop, other important findings such as thermal performance factor is very much essential to analyze the performance of any type of heat exchanger^67^. The TPF is established using integrated impact of flow and thermal characteristics and computed based on the Nusselt numbers and friction factors^15,30,63^to enhance the convective heat transfer. The TPF value should always be greater than unity for the efficient performance of heat exchanger^15,63^. The heat exchanger’s thermal performance factor was estimated using Eq. (13) and shown in Fig. 10. From the Fig. 10, it can be seen that the methanol and water-based hybrid nanofluids exhibit TPF higher than 1 for all concentrations and demonstrated noteworthy performance over the whole range of volume concentration and Re. On the other hand, Silicone oil-based hybrid nanofluids at 0.02% concentration exhibited a marginally deviating value from 1 at high Re. At 0.06% concentration, a maximum TPF value of 1.24 has been noticed as Re increases from 100 to 230 as illustrated in Fig. 10c. Castor oil-based hybrid nanofluids shows lower TPF (~ 0.997) at initial concentrations of 0.02 and 0.025 vol% and thereafter displays higher TPF (1.014–1.16)for 0.03–0.06 vol% hybrid nanofluids.

### Cost analysis

Cost analysis can be determined by considering weighted average capital, equipment installation, service, and maintenance costs^68^. According to the Hall method^69^, cost analysis was carried out by considering the HEX standards, flow characteristics, and framework of the material.

The heat exchanger’s overall cost is determined using the Hall approach using Eq. (18).

18 $$\:{\text{C}}_{\text{O}}=\:{\text{C}}_{\text{Y}\text{O}}+{\text{C}}_{\text{U}\text{I}}$$

where C_O_, C_YO,_ and C_UI_ are overall cost, yearly operational cost, and up-front investment cost.

The area of the heat exchanger affects the up-front investment cost, which may be estimated with the assistance Eq. (19)^69^.

19 $$\:{\text{C}}_{\text{U}\text{I}}={\text{a}}_{1}+({\text{A}}^{\text{a}_3}\times\:{\text{a}}_{2})$$

where A is heat exchanger’s area (m^2^).

For stainless steel material, a_1_, a_2_, and a_3_values were taken as 8000, 259.2, and 0.9 respectively^70^.

The entire yearly operational cost is calculated using Eq. (20) by summing up the cost of pumping power to compensate for pressure losses^69^.

20 $$\:{\text{C}}_{\text{Y}\text{O}}=\sum\:_{\text{K}=1}^{\text{n}}\frac{{\text{C}}_{\text{y}}}{{(1+\text{i})}^{\text{K}}}$$

where C_y_ refers to annual operation cost and it is estimated by Eq. (21)^69^,

21 $$\:{\text{C}}_{\text{y}}=\text{H}\times\:\text{P}\times\:{\text{C}}_{\text{u}}$$

where H and C_u_ stands for overall yearly operating hours and utility cost respectively^69^.

22 $$P = ~\frac{1}{\eta } \times \left( {\left( {\frac{{\dot{m}_{t} }}{{\rho _{t} }}} \right) \times \Delta P_{t} + \left( {\frac{{\dot{m}_{s} }}{{\rho _{s} }}} \right) \times \Delta P_{s} } \right)$$

where ṁ, ΔP, ρ, and η represents mass flow rate (kg/s), pressure drop (Pa), density, and pump efficiency (70%), subscripts t and s represent tube side and shell side respectively.

The interest rate (i), the lifespan of the project (n), and overall yearly operating hours (H) and utility cost (C_u_)are 25%, 5 years,2190 h/year, and 0.12 respectively. For ordinary industries, the average rate of interest, “i,” is considered as 25%.

To determine the overall cost of the heat exchanger, the following experimental data, which is displayed in Table 9, is considered and incorporated into the aforementioned calculations.

**Table 9:** Table displaying the data collected during experiments, fluid characteristics, and overall cost.

S.No	The property measured from experiments	Corresponding value
1.	Hot water mass flow rate in tube side ($\dot{m}_{t}$)	0.00611 kg/s
2.	Pressure drop in tube side ($\:{\Delta\:}{\text{P}}_{\text{t}}$)	11.07 Pa
3.	Hot water density ($\:{{\uprho\:}}_{\text{t}})$	988.04 kg/m^3^
4.	Hybrid nanofluids mass flow rate in shell side ($\dot{m}_{s}$)	0.01812 kg/s
5.	Pressure drop in shell side ($\:{\Delta\:}{\text{P}}_{s}$)	129.52 Pa
6.	Hybrid nanofluid density (($\:{{\uprho\:}}_{\text{s}})$	1001.99 kg/m^3^

Using the above data shown in Table 6, the overall cost of a double pipe heat exchanger is found to be $ 8730.

###  Comparison of heat transfer metrics from the current work with previously conducted studies

Table 10 displays a comparison of various convective heat transfer parameters of the current study with those of previous investigations. In comparison to other hybrid nanofluids, the Cu-MXene hybrid nanofluids utilized in the current work possess higher enhancement in heat transfer capacities especially for methanol-based and water-based at very low concentrations ranging from 0.02 to 0.06%. This is probably due to excellent stability and thermal conductivity of hybrid nanofluids.

**Table 10:** Table distinguishing the work that is being carried out presently with literature.

References	HybNP / NP	Concentration (Wt/vol%)	Base fluid	Flow regime	Maximum enhancement (%)					TPF
					Nu	h	f	ΔP	U	
^15^	Fe_3_O_4_ - SiO_2_	0.2–1.0	Water	Laminar	25	21	37	23	24	1.18
^27^	Al_2_O_3_ -CuO	0.6–1.8	Distilled water	Turbulent	43	11	31	19	–	1.7
^30^	CoFe_2_O_4−_BaTiO_3_	0–1.0	EG	Laminar	22.2	32	21	–	–	1.42
^59^	Cu– GO	0.3	Therminol VP – 1	Turbulent	211	–	242	–	–	1.44
^63^	Al_2_O_3_ –Cu	Simulation	Water	Turbulent	58	14	4.5	10	–	1.12
^66^	CuO–ZnO	0.01	Water	Laminar to turbulent	33	7	–	13	–	1.45
^71^	Cuo-Mgo-TiO_2_	0.1–0.5	Water	Laminar	–	46	85	41	10	–
^72^	Al_2_O_3_ –Cu	0.1	Water	Laminar	13.5	24	16	–	–	–
Current work	Cu-MXene	0.02–0.06	Methanol, Water, Silicone oil, and Castor oil	Laminar	39–80	56–216	28–108	31–109	70–156	1.15–1.41

### Uncertainty analysis

Each investigation was carried out multiple times with hybrid nanofluids and their base fluids. In general, the accuracy of the experimental results relies on how precisely every approach and measuring instrument is being carried out. The uncertainty of the parameters can be assessed utilizing the root sum square combination of the effects of each individual input in addition to Moffat’s error propagation^73^, specifically developed by Kline & McClintock^74^. Tables 11 and 12 provides the details of the major parameters’ maximum error and uncertainty associated with each experimental run.

Equations (23–27) are used to figure out the uncertainties related to Re, Q, Nu, h, and *f*^75^. The previously published investigation shows the uncertainty values of the ρ, µ, and k respectively^54^.

The uncertainty calculations for water-based Cu-MXene hybrid nanofluids at 0.02 vol% is shown below.

For heat transfer rate, uncertainty is calculated as below.

23 $$u_{{,Qc}} = \sqrt {\left( {\frac{{\Delta T}}{T}} \right)^{2} + \left( {\frac{{\Delta \dot{m}_{c} }}{{\dot{m}_{c} }}} \right)^{2} + \left( {\frac{{\Delta C_{{pc}} }}{{C_{{pc}} }}} \right)^{2} }$$

$$\:\:\:\:\:\:\:\:\:\:\:=\:\:\:\sqrt{{\left(0.01\right)}^{2}+{{\left(0.025\right)}^{2}+\left(0\right)}^{2}}$$= 0.027%

For the Nusselt number, uncertainty is calculated as below.

24 $$\:{u}_{,Nu}=\:\sqrt{{\left(\frac{\varDelta\:{D}_{h}}{{D}_{h}}\right)}^{2}+{\left(\frac{\varDelta\:k}{k}\right)}^{2}+{\left(\frac{\varDelta\:h}{h}\right)}^{2}}$$

= $$\:\sqrt{{{\left(0\right)}^{2}+{\left(0.005\right)}^{2}+\left(0.008\right)}^{2}}$$ = 0.0094%

For heat transfer coefficient (h), uncertainty is calculated as below.

25 $$\:{u}_{,h}=\:\sqrt{{\left(\frac{\varDelta\:T}{T}\right)}^{2}+{\left(\frac{\varDelta\:Q}{Q}\right)}^{2}+{\left(\frac{\varDelta\:A}{A}\right)}^{2}}$$

$$\:\:=\:\:\:\sqrt{{{\left(0.011\right)}^{2}+{\left(0.008\right)}^{2}+\left(0\right)}^{2}}$$= 0.0136%

For the Reynolds number (Re), uncertainty is calculated as below.

26 $$\:{u}_{,Re}=\:\sqrt{{{\left(\frac{\varDelta\:\rho\:}{\rho\:}\right)}^{2}+{\left(\frac{\varDelta\:\mu\:}{\mu\:}\right)}^{2}+\left(\frac{\varDelta\:{D}_{h}}{{D}_{h}}\right)}^{2}+{\left(\frac{\varDelta\:v}{v}\right)}^{2}}$$

= $$\:\sqrt{{{\left(0.00011\right)}^{2}+\left(0.0562\right)}^{2}+{\left(0\right)}^{2}+{\left(0.0077\right)}^{2}}$$ = 0.0567%

For friction factor (*f*), uncertainty is calculated as below.

27 $$\:{u}_{,f}=\:\sqrt{{{\left(\frac{\varDelta\:L}{L}\right)}^{2}+\left(\frac{\varDelta\:{D}_{h}}{{D}_{h}}\right)}^{2}+{\left(\frac{\varDelta\:v}{v}\right)}^{2}+{\left(\frac{\varDelta\:\rho\:}{\rho\:}\right)}^{2}+{\left(\frac{\varDelta\:P}{P}\right)}^{2}}$$

= $$\:\sqrt{{{\left(0\right)}^{2}+{\left(0\right)}^{2}+{\left(0.0077\right)}^{2}+\left(0.00011\right)}^{2}+\:{\left(0.00166\right)}^{2}}$$ = 0.00788%

**Table 11:** Table indicating instrument error.

Measured parameters	Error
Fluid temperatures	
Hot fluid inlet (h_i_)	± 0.2 °C
Hot fluid outlet (h_o_)	± 0.2 °C
Inlet cold fluid (C_i_)	± 0.2 °C
Outlet cold fluid (C_o_)	± 0.2 °C
Flow rate measured	± 0.3 ml/s
Time measurement for steady state	± 3 s
Circulation speed for peristaltic pump	± 1 RPM

**Table 12:** Table indicating shell side uncertainty analysis.

Variables	Uncertainty in %
Reynolds number (Re)	2.46
Heat transfer rate (Q)	3.24
Nusselt number (Nu)	0.86
Heat transfer coefficient (h)	0.75
Darcy Friction factor (*f*)	0.55

### Economic and environmental benefits of Cu-MXene hybrid nanofluids

Copper is a noble and reasonably priced metal that may be used in many applications as a substitute for the costliest noble metals like gold and silver due to its unique properties. Furthermore, copper nanoparticles are considered capable thermal fluids that may replace traditional fluids in cooling applications^76^. Similarly, MXenes are also considered a quickly evolving family of materials comprising 2D layers and have remarkable thermal characteristics^77^ and nontoxic nature^78^. Hybrid nanofluids containing nanoparticles of Cu and MXene in base fluids improve the thermal conductivity of convectional fluids which further enhances heat transfer efficiency thereby reducing operational cost, size of the heat exchanger equipment, power consumption, and amount of coolant requirement. Table 13, indicates the thermal conductivity values of common coolants utilized in industries.

**Table 13:** Table representing the thermal conductivity of common coolants or base fluids.

Base fluids/traditional coolants	Thermal conductivity (W/m. K)
Water	0.598
Castor oil	0.177
Silicon oil	0.151
Methanol	0.202
Ethylene glycol	0.258
Mineral oil	0.136
Dynalene HC-FGat 20 °C	0.281
Propylene Glycol (30%)	0.243
Copper	401
MXene	~ 20

### Limitations and future work

The major limitation of the current study can be the scalability challenges associated with the large-scale processes. In addition, the oxidation behavior of copper is another major concern that will influence the thermophysical properties of hybrid nanofluids. Hence, thorough and detailed strategies need to be framed to prevent possible oxidation for longer usage and storage. Investigation of Cu-MXene hybrid nanofluids for corrosion in DPHE is also another challenging ask and can be considered as a possible future scope. Further enhancement of heat transfer in DPHE can also be conducted by developing hybrid nanofluids using commercial coolants such as radiator coolants, EG, etc. Moreover, a predictive model can be developed using various software’s like Ansys Fluent, COMSOL.

## Conclusion

Cu-MXene hybrid nanofluids were successfully synthesized using various base fluids to study the heat transfer performance of a modular double pipe heat exchanger. The convective heat transfer parameters such as Q, LMTD, Nu, h, ∆P, *f*, and TPF were determined through experimental approaches and validated using Aspen HYSYS simulation software for all concentrations of hybrid nanoparticles.

The heat transfer coefficient (h) was successfully improved for all Cu-MXene based hybrid nanofluids compared to their base fluids by 216%, 146%, 60%, and 82% for methanol-based, water-based, silicone oil-based, and castor oil-based Cu-MXene hybrid nanofluids respectively. Enhancement of pressure drop (ΔP) with 65%, 110%, 31%, and 56% was observed for water-based, methanol-based, castor oil-based, and silicone oil-based Cu-MXene hybrid nanofluids respectively. The thermal performance factor (TPF) values were reported to be greater than one, especially for methanol-based and water-based Cu-MXene hybrid nanofluids with 1.414 and 1.412 respectively, which indicates improved performance of heat transfer in DPHE. Finally, Cu-MXene hybrid nanofluids synthesized using water-based, methanol-based, castor oil-based, and silicon oil-based are highly recommended for cooling or heating applications and high temperature lubrications that are applicable either in electronics or industrial appliances under laminar flow conditions.

## Electronic supplementary material

Below is the link to the electronic supplementary material.

Supplementary Material 1

Supplementary Material 2

## Data Availability

Data is provided within the manuscript or supplementary information files.

## List of symbols

DPHE: Double pipe heat exchanger
ID: Inner diameter (m)
OD: Outer diameter (m)
D_i_: Inner tube diameter (m)
D_io_: Inner tube outer diameter (m)
D_o_: Outer shell inner diameter (m)
D_oo_: Outer shell outer diameter (m)
A: Pipe cross-sectional area (m^2^)
d_h_: Hydraulic diameter (m)
L: Length of heat exchanger (m)
NRTL: Non-Random Two-Liquid model
P: Wetted perimeter (m)
LMTD: Log mean temperature difference
TPF: Thermal performance factor
Q: Heat transfer rate (W)
Cu: Copper
SDS: Sodium dodecyl sulfate
RPM: Revolutions per minute (r.min^− 1^)
PID: Proportional Integral Derivative
H: Heat transfer coefficient (W/m^2^. K)
ṁ: Mass flow rate (kg/s)
Cp: Specific heat (J/kg. K)
Nu: Nusselt number ($\:\frac{h\:\times\:\:d}{k})$
Pr: Prandtl number $\:\left(\frac{{C}_{p}\times\:\:\mu\:}{k}\right)$
Re: Reynolds number $\:\left(\frac{D\times\:\rho\:\times\:v}{\mu\:}\right)$
T: Temperature (°C)
HEX: Heat exchanger
t: Time (s)
*v*: Fluid velocity (m/s)
k: Thermal conductivity (W/m. K)
wt: Weight (g)
v/c: Volume concentration
vol%: Volume concentration (%)
w: DPHE at wall conditions
$$\:u$$: Uncertainty (%)

## Greek symbols

Ф: Volume concentration
µ: Dynamic viscosity (Pa.s)
ƒ: Friction factor
ρ: Density (kg/m^3^)
ɛ: Effectiveness
ΔT: Temperature difference (°C)
ΔP: Pressure drop (Pa$\:)$

## Subscripts

$$\:{h}_{f}$$: Hot fluid
h_ybnf_: Hybrid nanofluids
b: Bulk
f: Fluid
i: Inner
o: Outlet
bf: Base fluids
c: Cold fluid
avg: Average
