# Supplementary material for: Novel Cu-MXene hybrid nanofluids for the experimental investigation of thermal performance in double pipe heat exchanger
Source: Sci Rep. 2025 Mar 22;15:9937. doi: 10.1038/s41598-025-94330-5 (PMC11929880; doi:10.1038/s41598-025-94330-5)
Supplement: Supplementary file 1 — Supplementary Material 1 [file 41598_2025_94330_MOESM1_ESM.docx]

**Novel Cu-MXene Hybrid Nanofluids for the Experimental Investigation of Thermal Performance in Double Pipe Heat Exchanger**

Kodi Rajesh Kumar, Mohammed Rehaan Chandan, Bandaru Kiran and Aabid Hussain Shaik*

Colloids and Polymers Research Group

School of Chemical Engineering

Vellore Institute of Technology, Vellore, Tamilnadu-632014

Corresponding author email: [aabidhussain.s@vit.ac.in](mailto:aabidhussain.s@vit.ac.in)


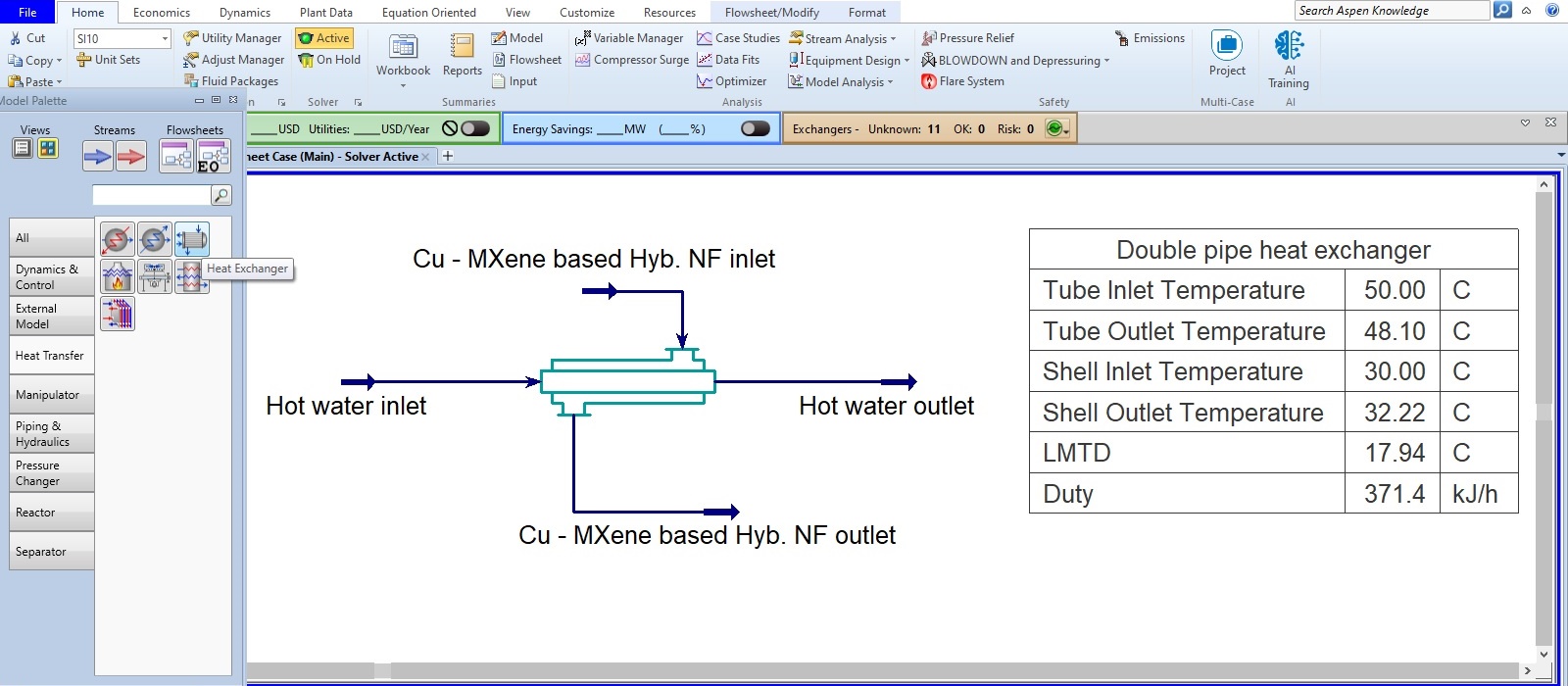


**Fig. S1.** Aspen HYSYS simulation model of DPHE.


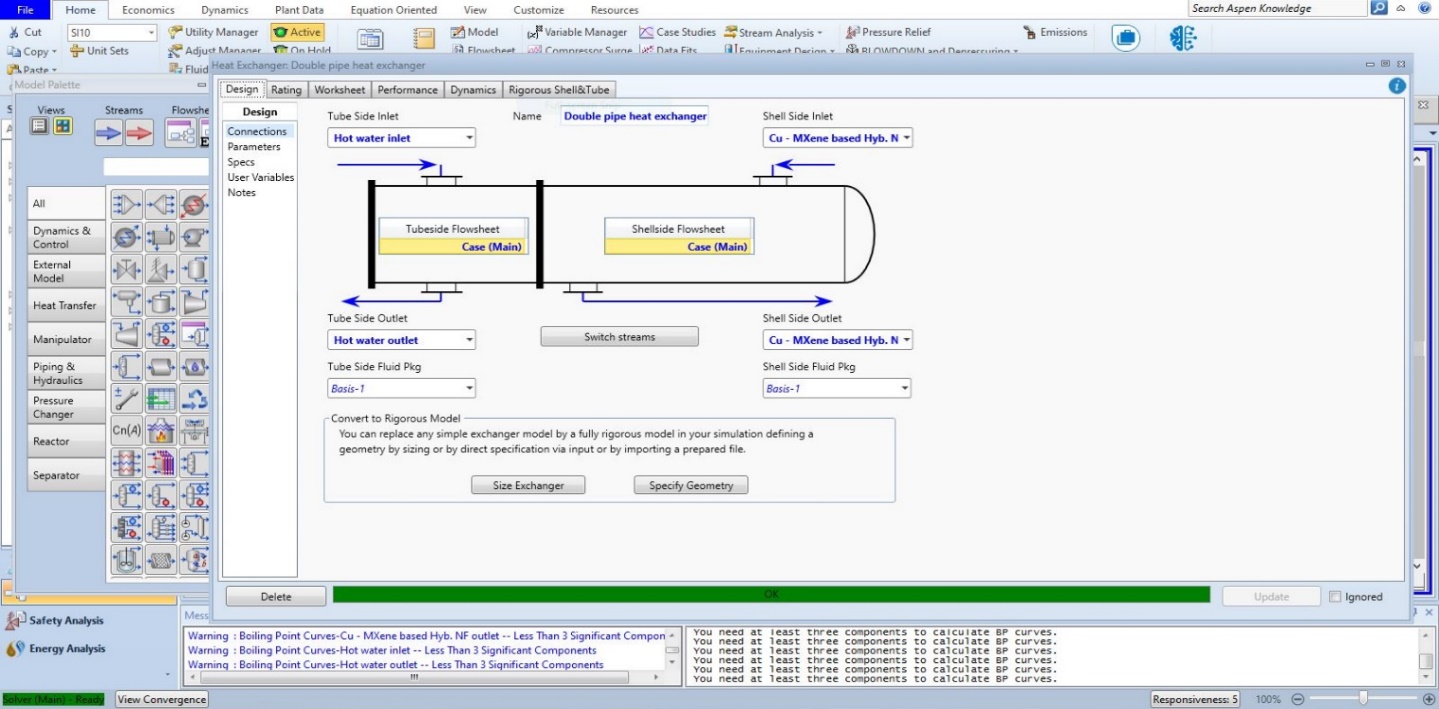


**Fig. S2.** Design of DPHE by using Aspen HYSYS.


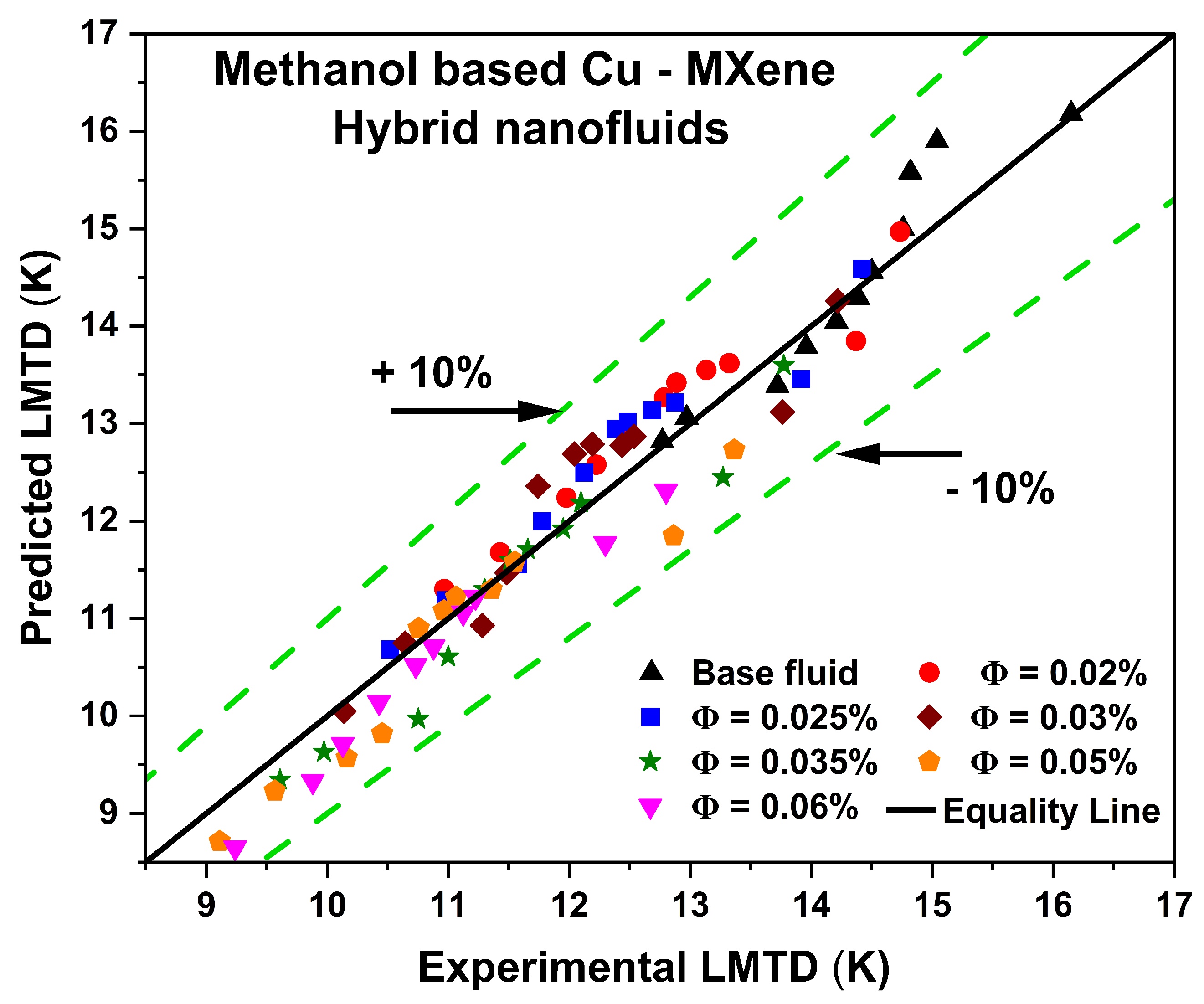

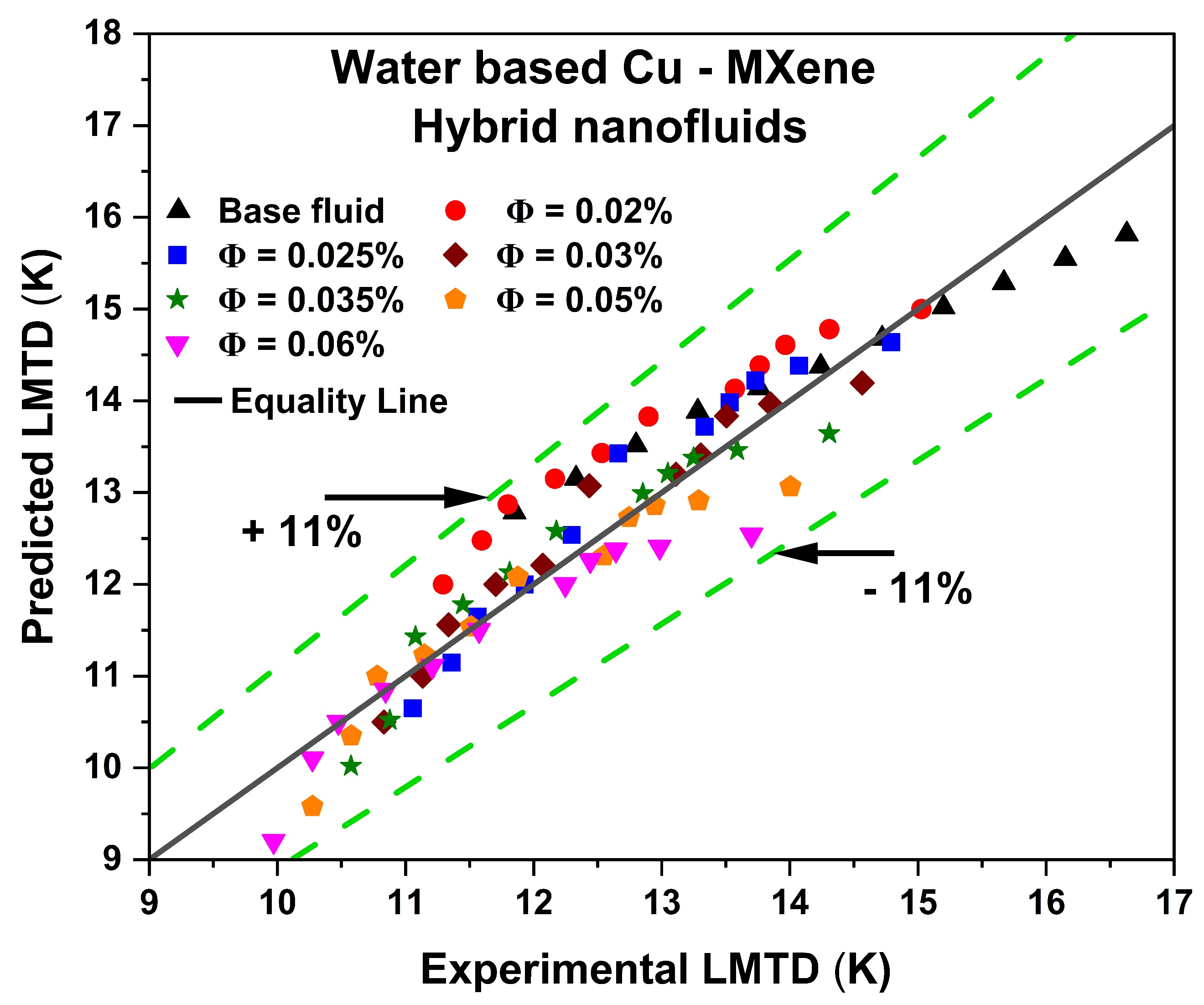


**(b**)

**(a**)


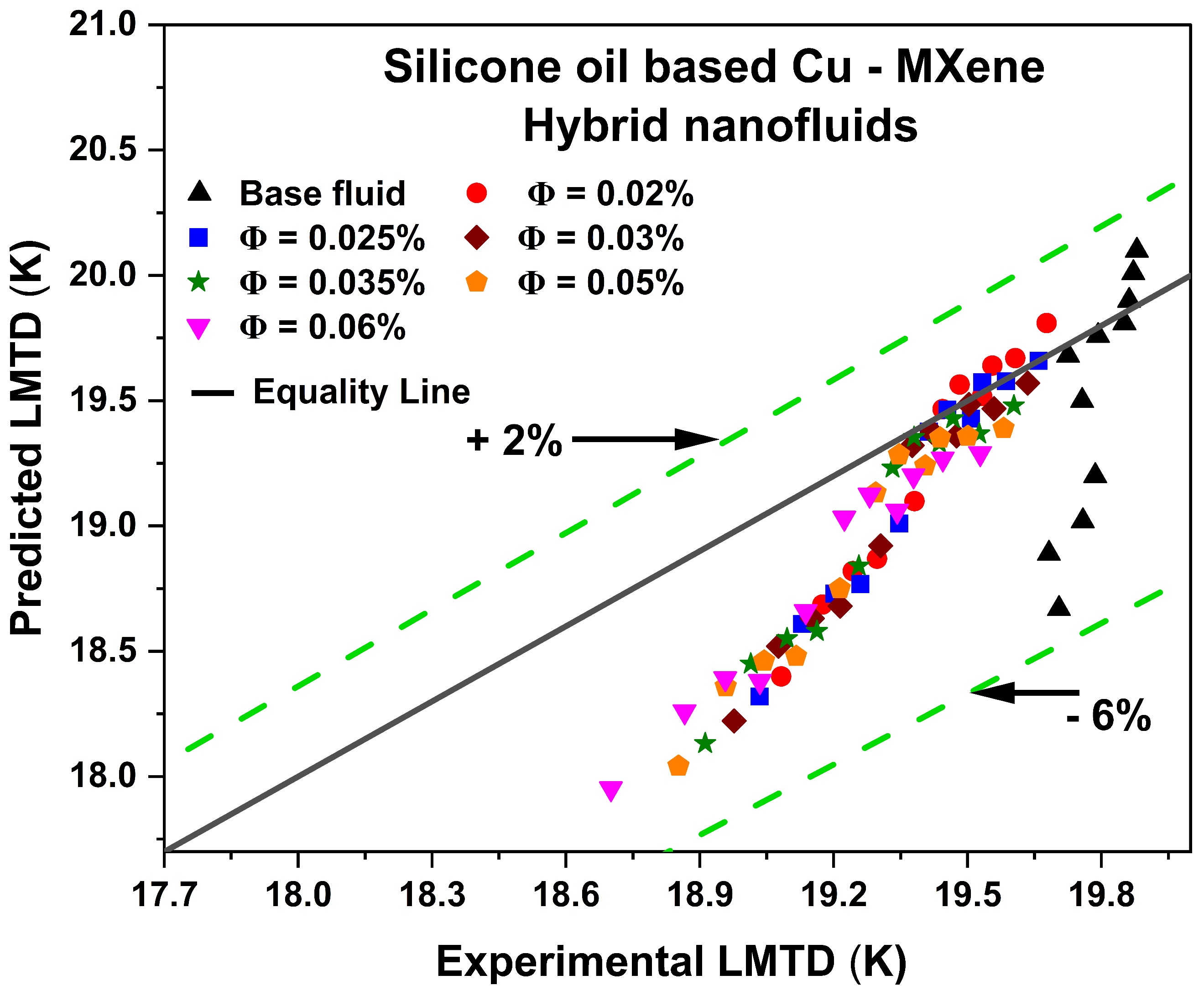

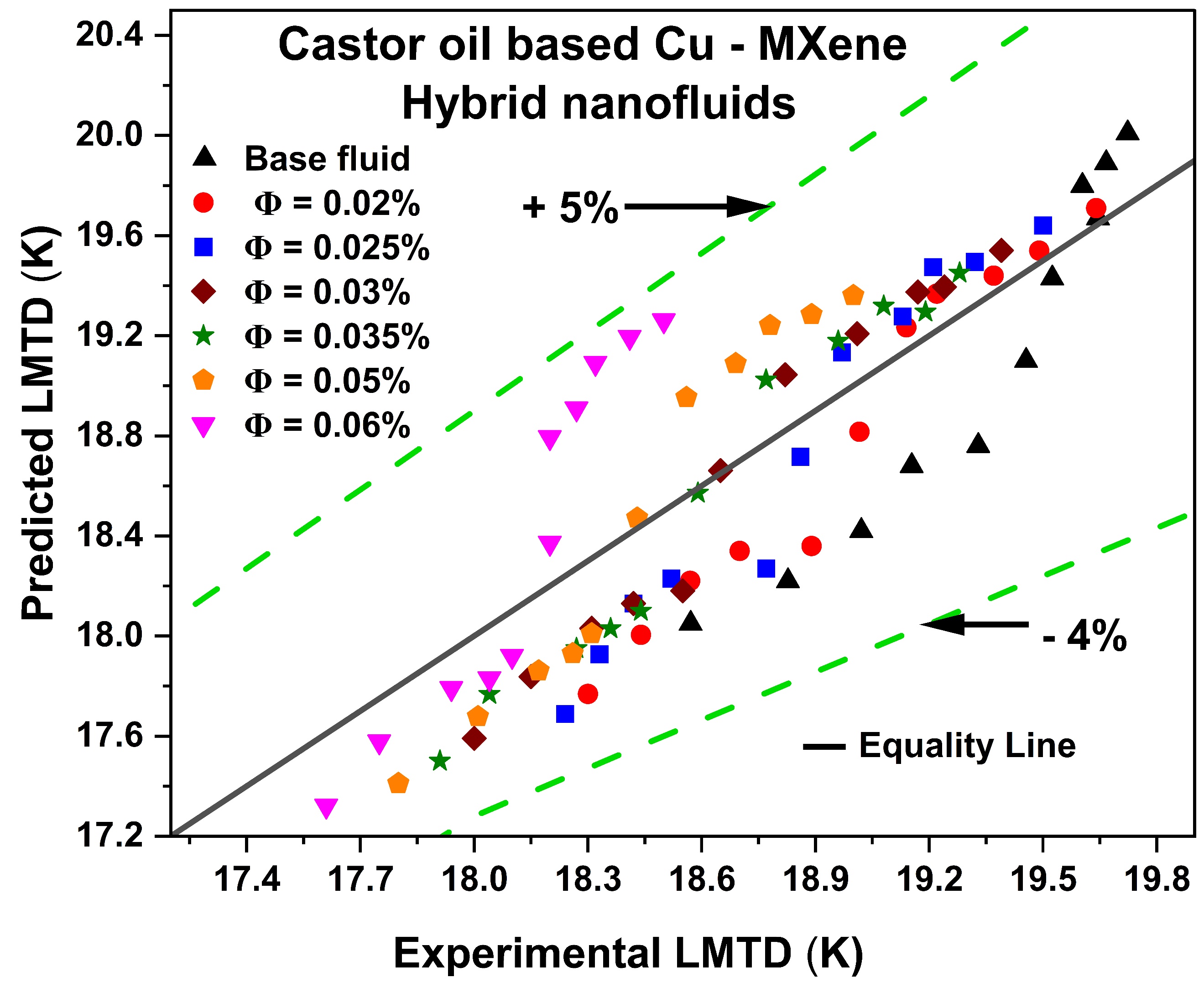


**(d**)

**(c**)

**Fig. S3:** Validation of LMTD using Aspen HYSYS simulation (a) methanol-based, (b) water-based, (c) silicone oil-based and, (d) castor oil-based Cu-MXene hybrid nanofluids**.**


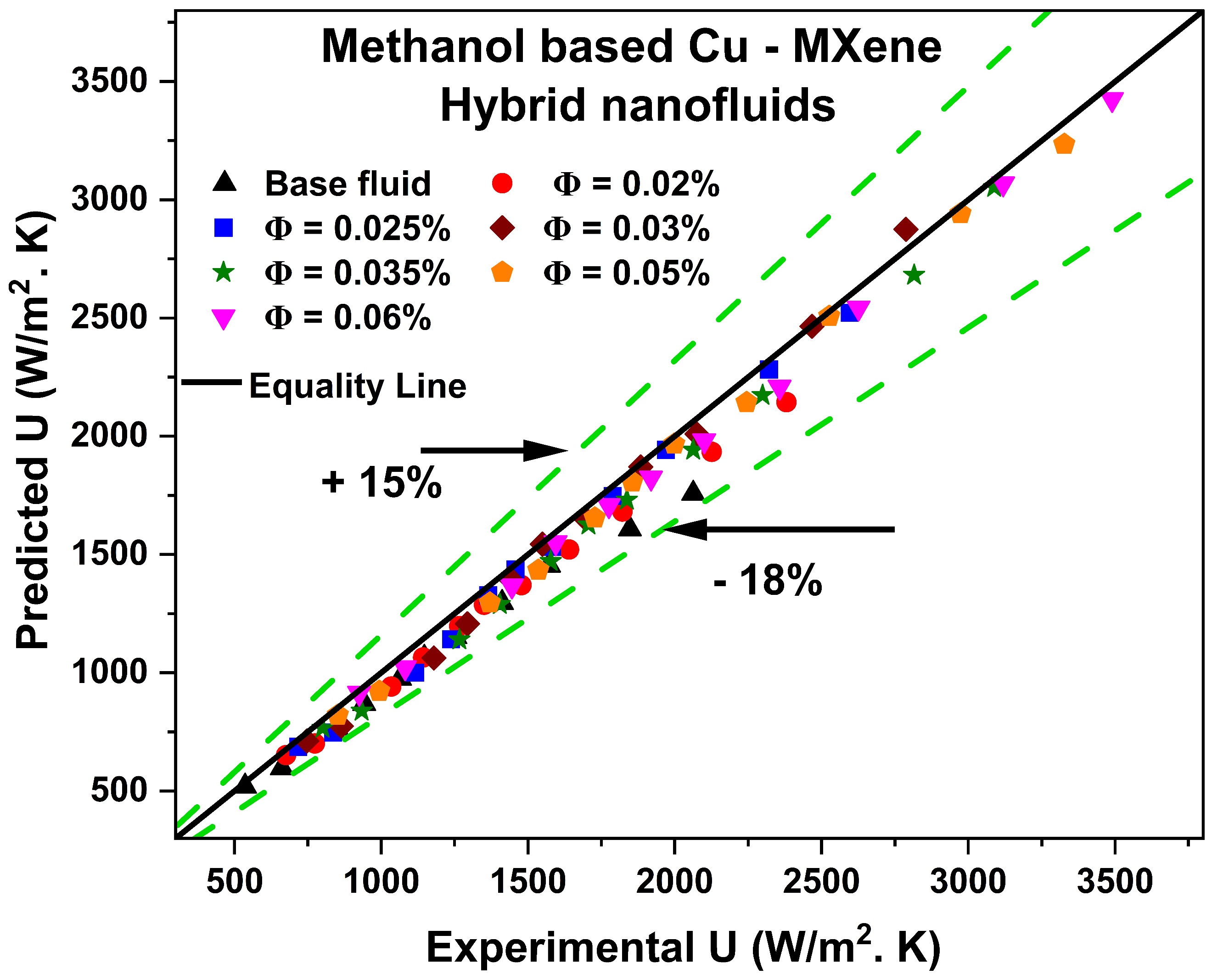

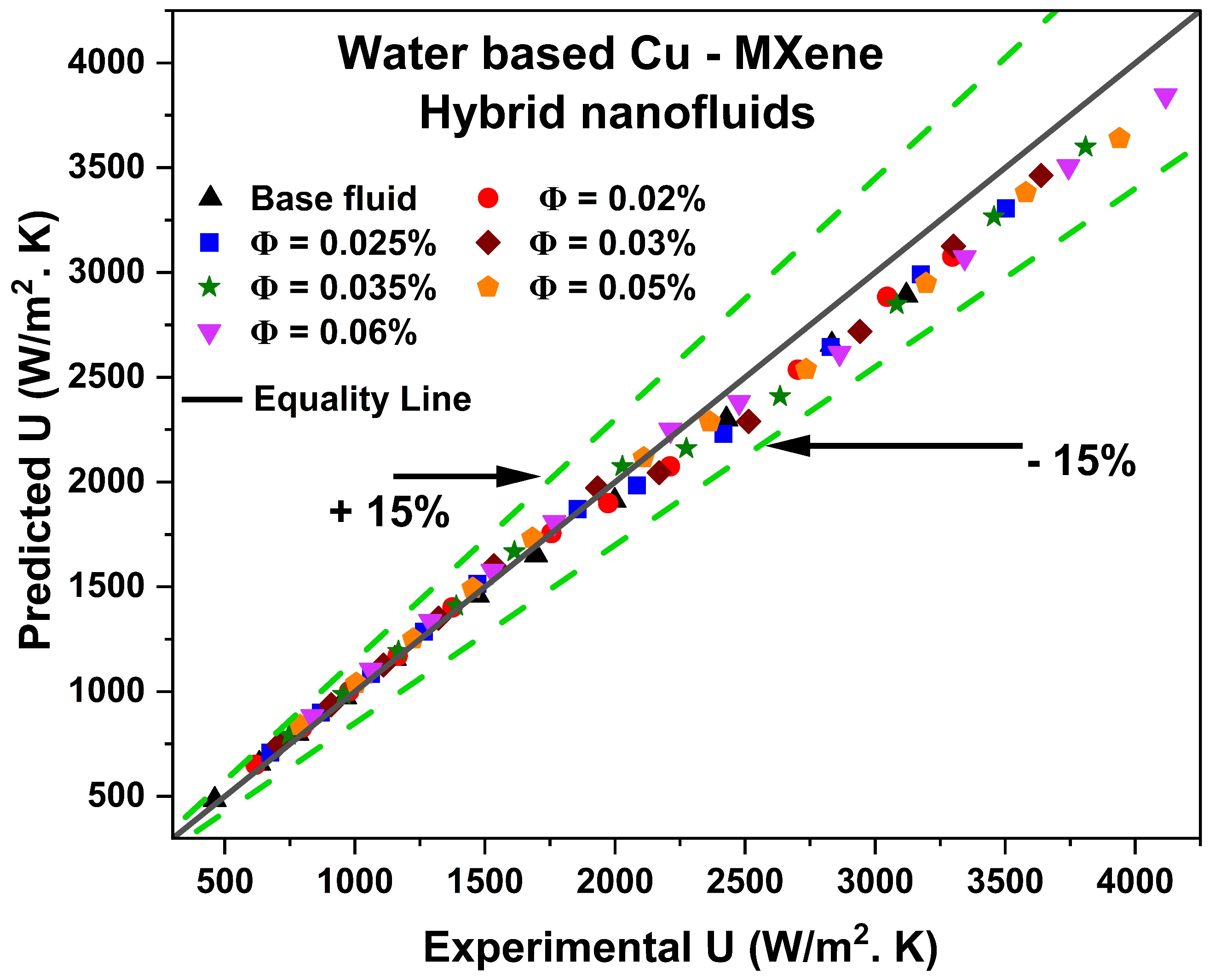


**(a**)

**(b**)


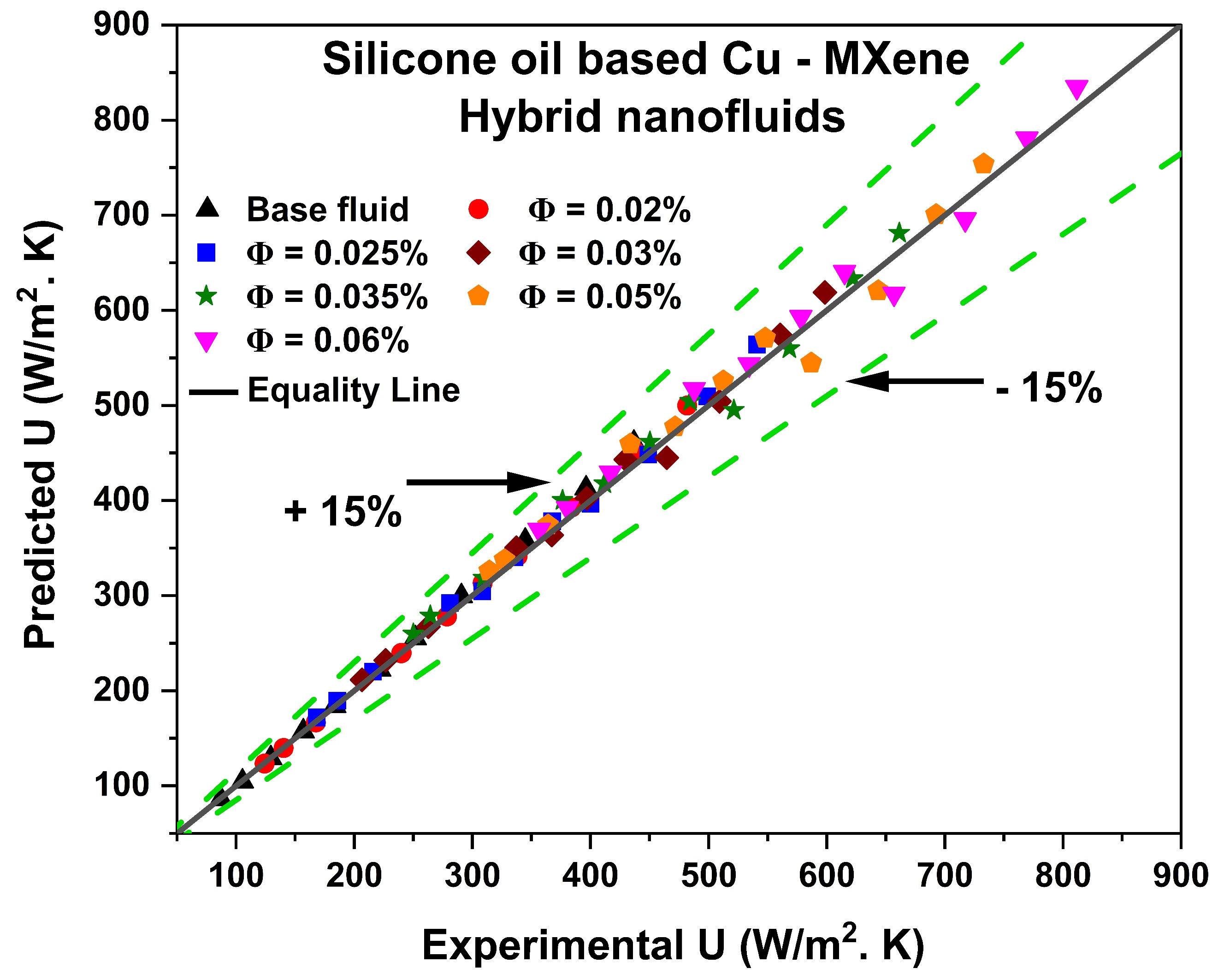

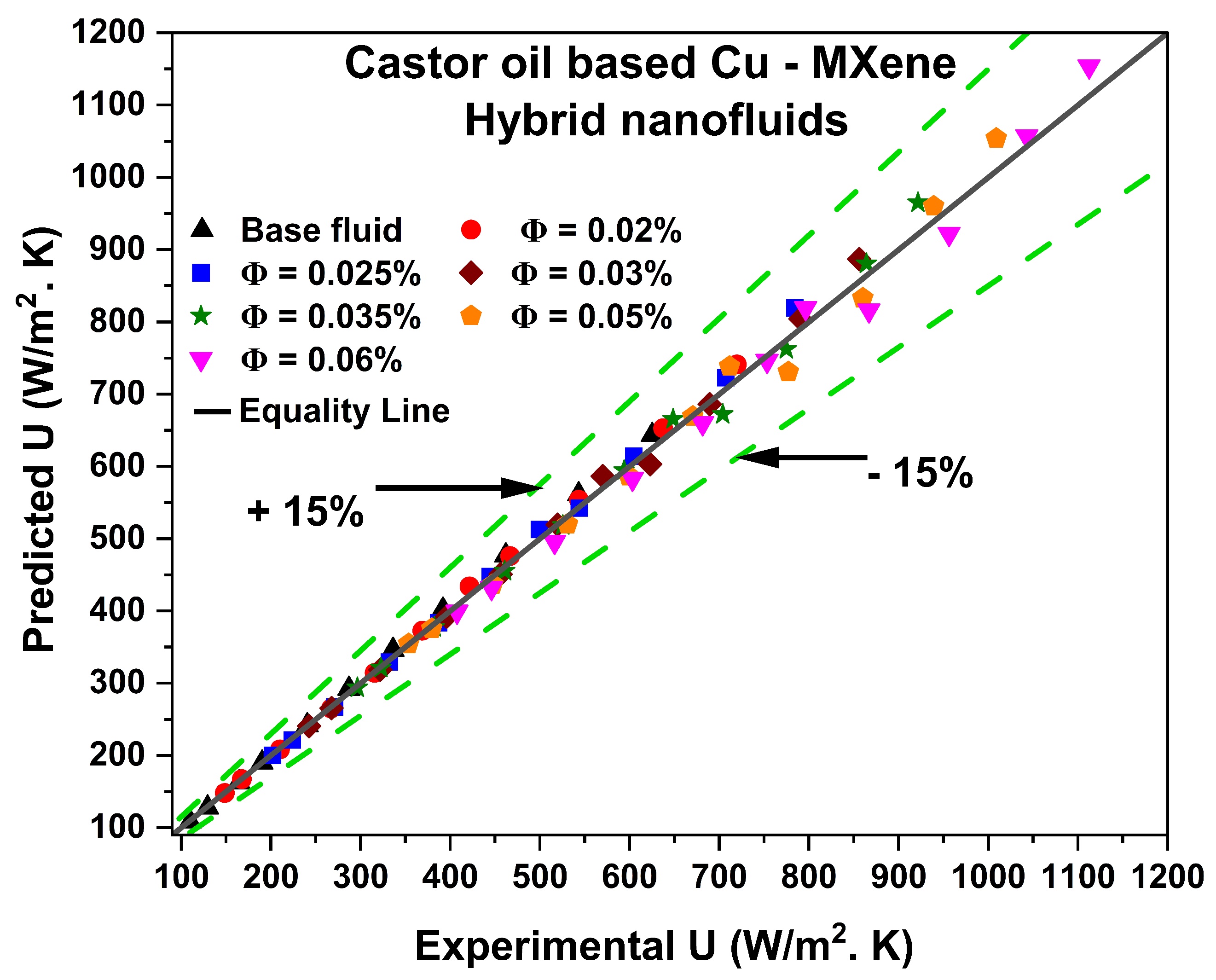


**(c**)

**(d**)

**Fig. S4:** Validation of U using Aspen HYSYS simulation (a) methanol-based, (b) water-based, (c) silicone oil-based and, (d) castor oil-based Cu-MXene hybrid nanofluids.


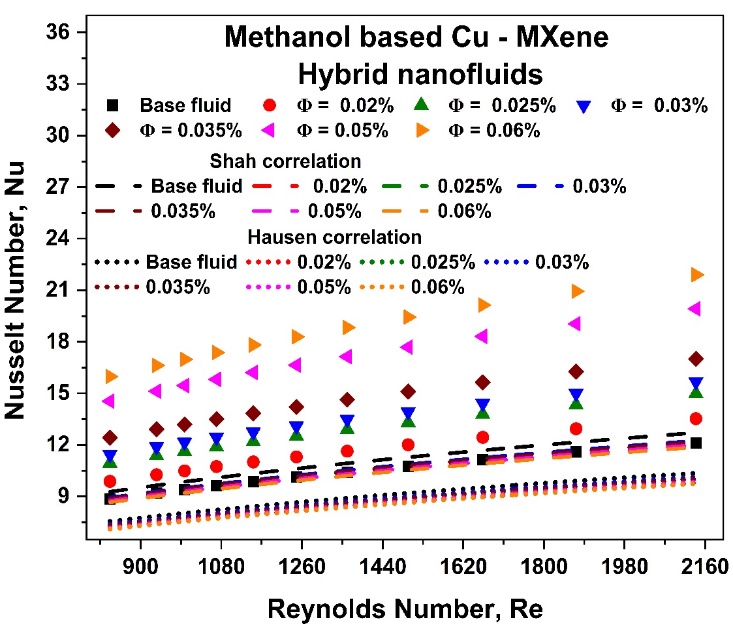

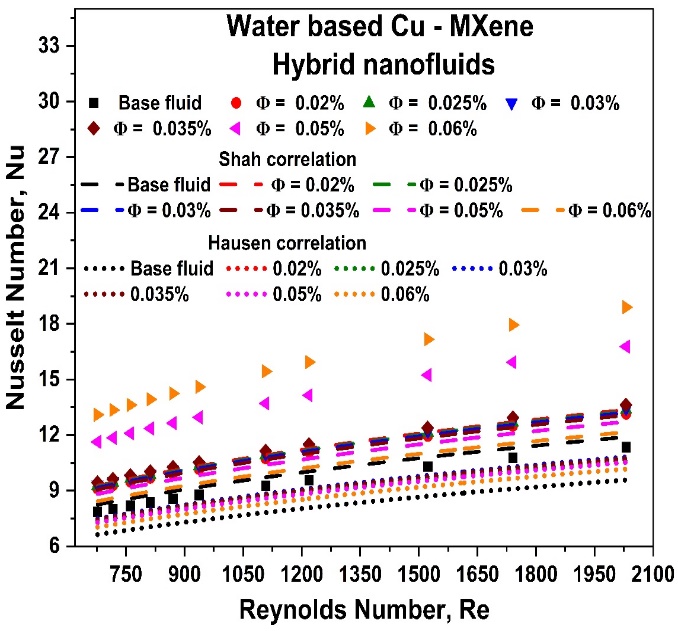

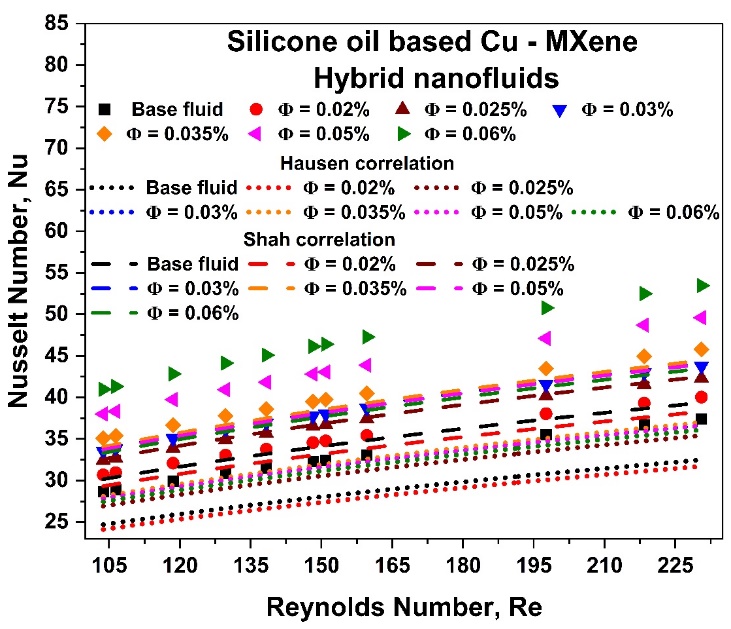

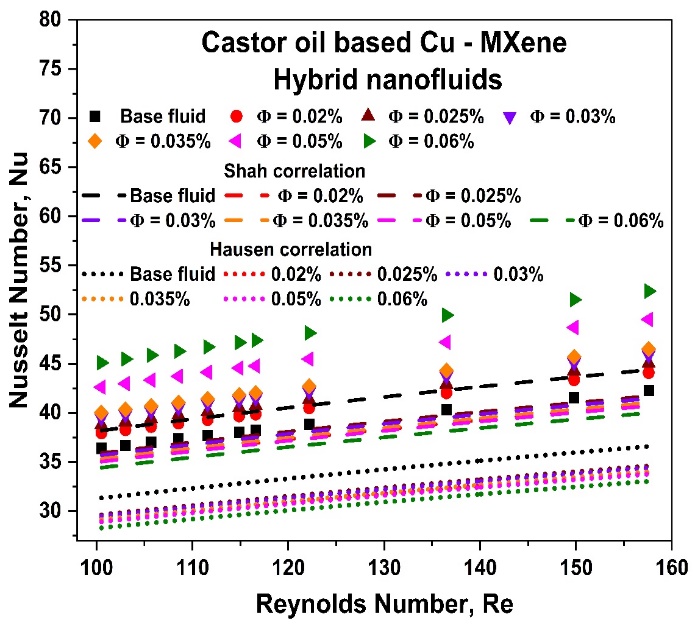


**(d**)

**(c**)

**(b**)

**(a**)

**Fig. S5.** Using correlations to validate the experimental Nusselt number for (a) methanol-based, (b) water-based, (c) silicone oil-based and, (d) castor oil-based Cu-MXene hybrid nanofluids.


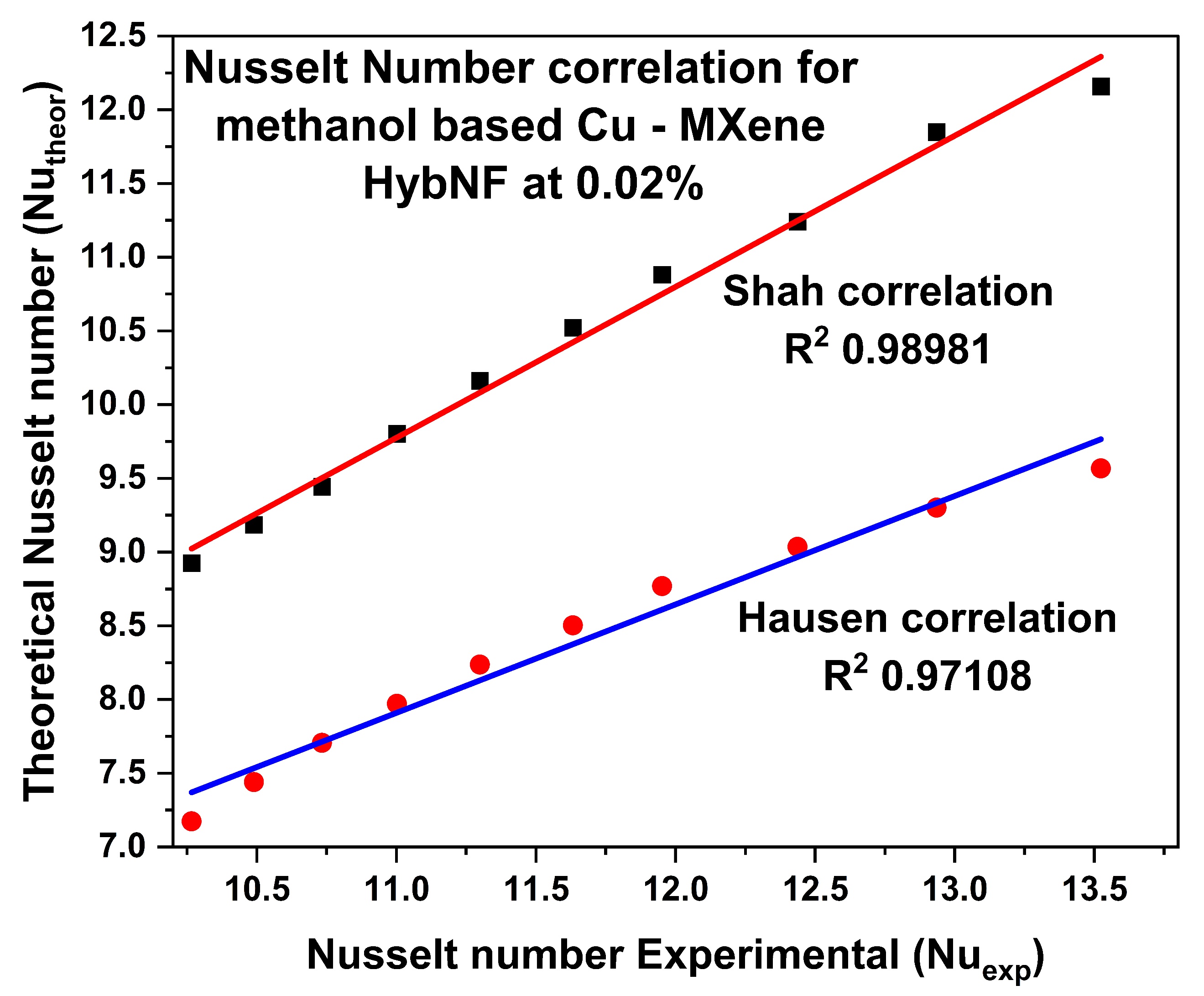

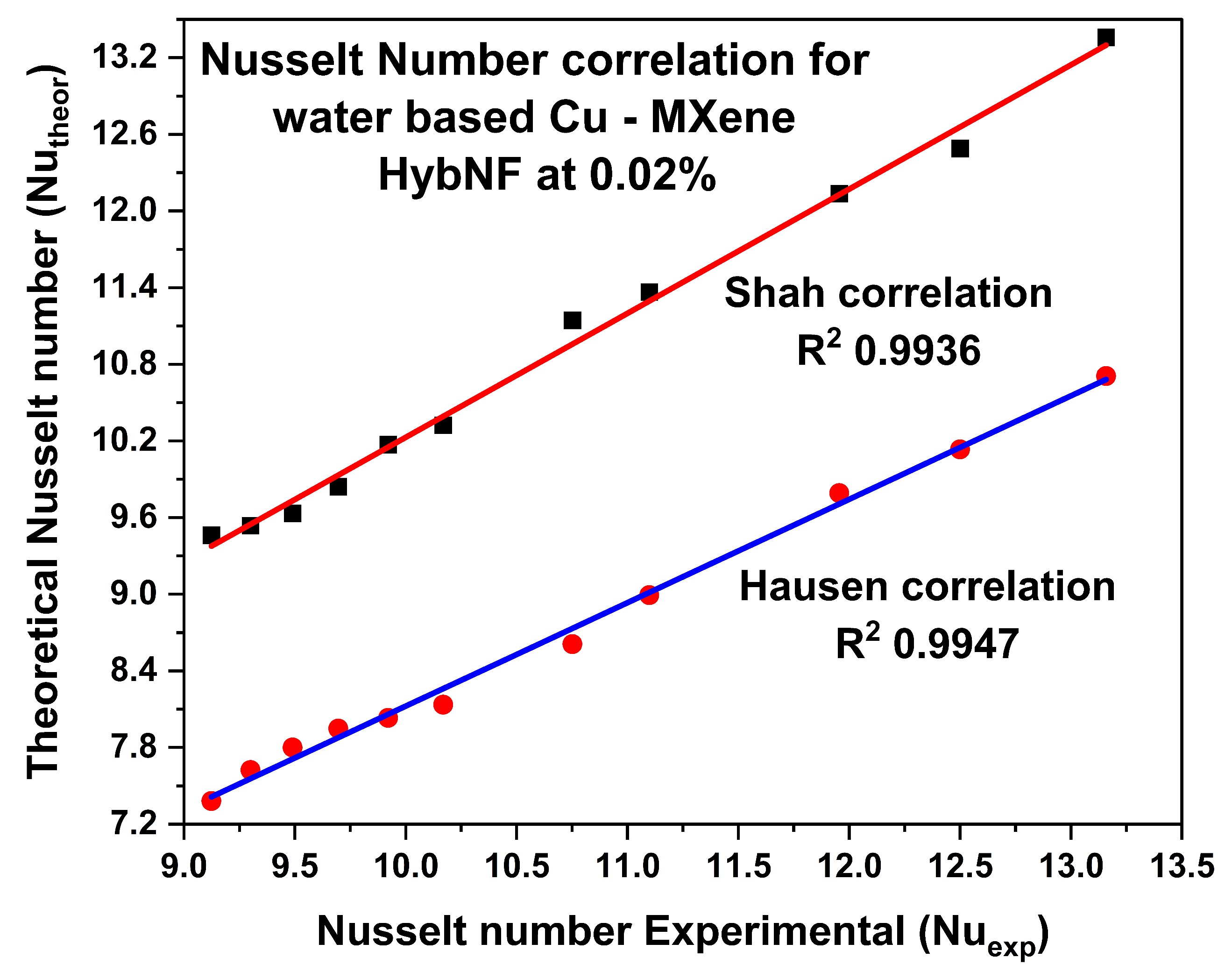

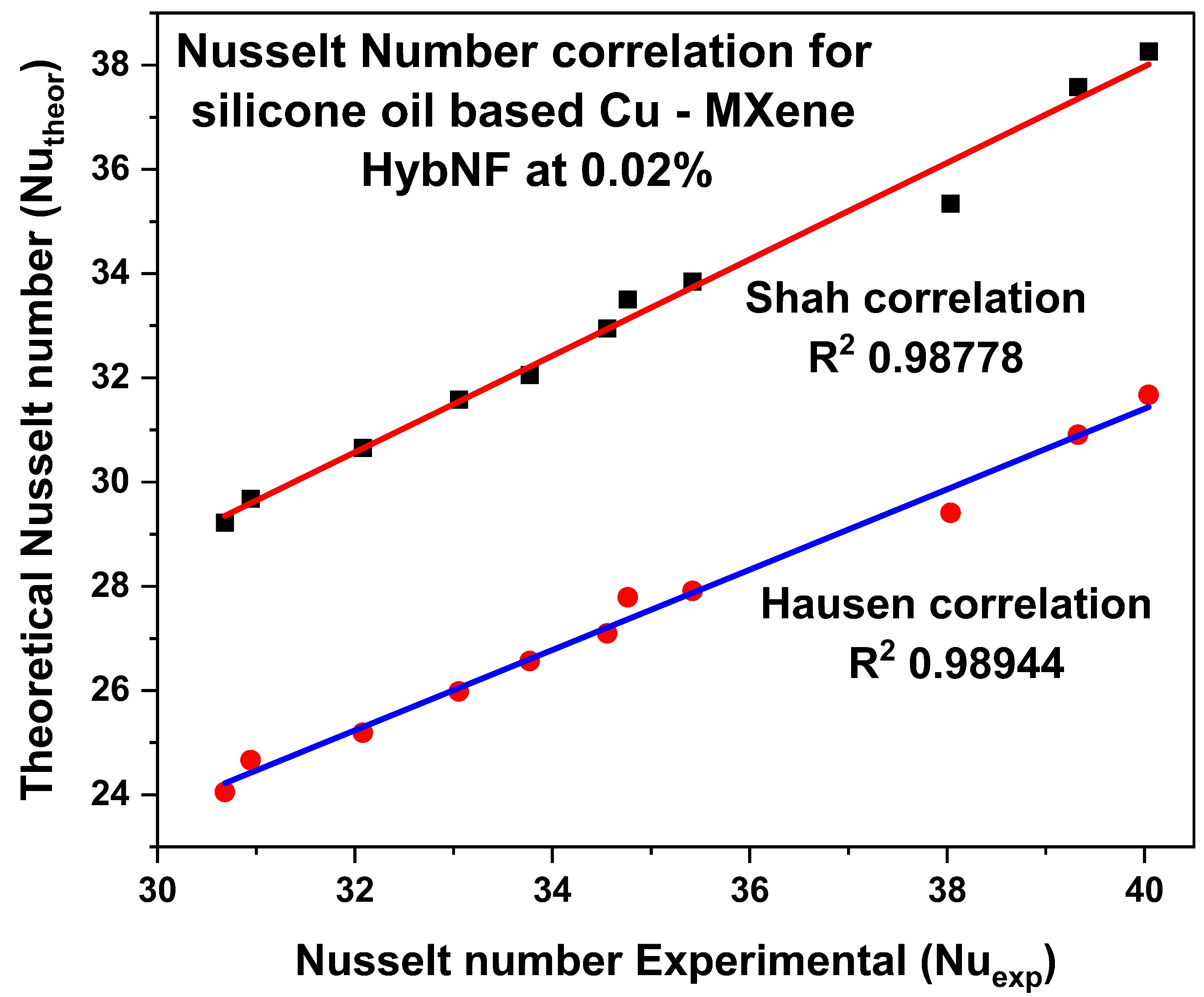

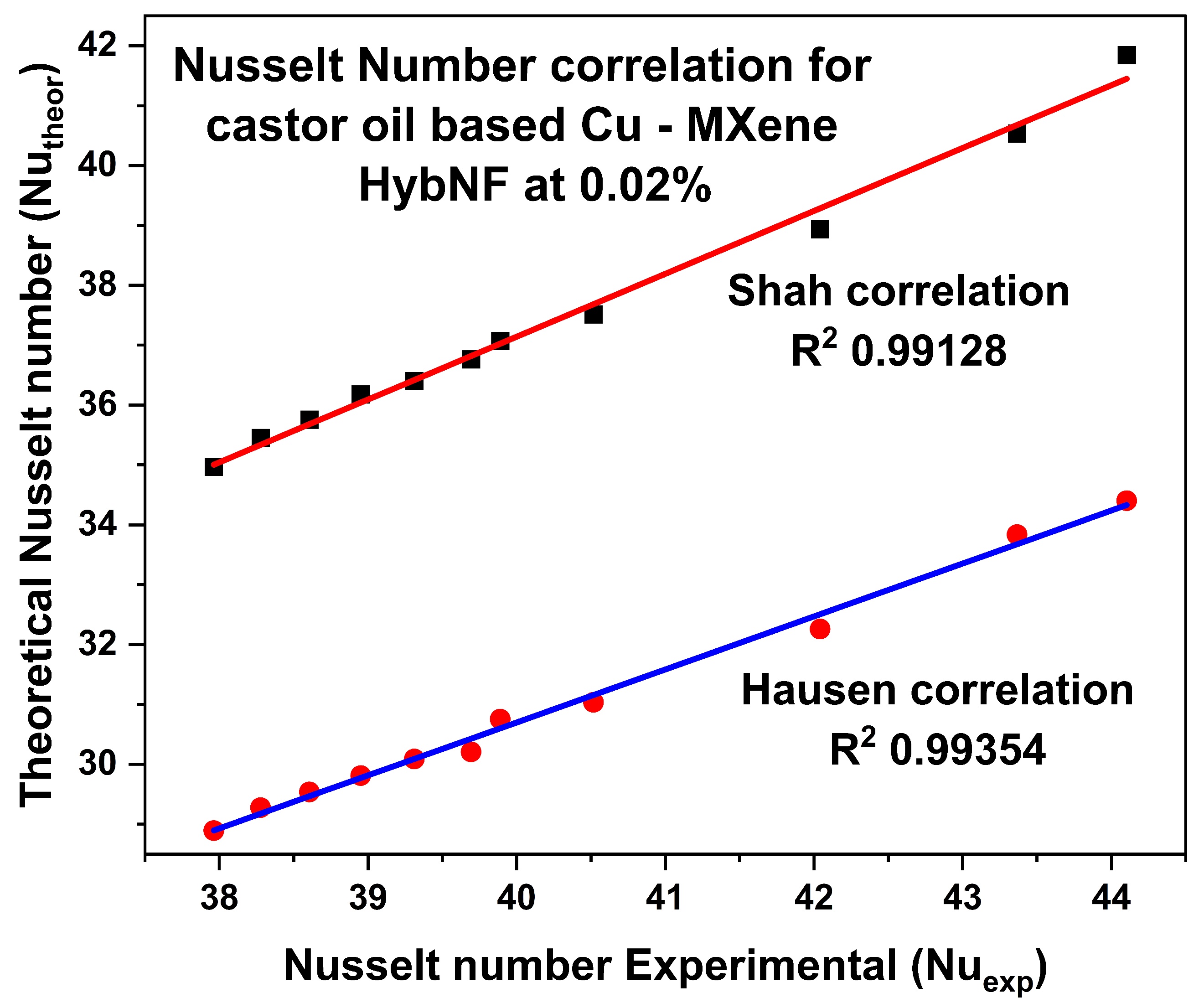


**(a**)

**(b**)

**(c**)

**(d**)

**Fig. S6.** R^2^ values for experimental Nusselt number Vs correlated Nusselt number for (a) methanol-based, (b) water-based, (c) silicone oil-based and, (d) castor oil-based Cu-MXene hybrid nanofluids for 0.02%.
