# Supplementary material for: Novel Cu-MXene hybrid nanofluids for the experimental investigation of thermal performance in double pipe heat exchanger
Source: Sci Rep. 2025 Mar 22;15:9937. doi: 10.1038/s41598-025-94330-5 (PMC11929880; doi:10.1038/s41598-025-94330-5)
Supplement: Supplementary file 2 — Supplementary Material 2 [file 41598_2025_94330_MOESM2_ESM.docx]

1. **Appendix**
2. The hydraulic diameter of inner tube (d_hi_) is same as its diameter i.e., $d_{hi}=D_{i}$, but for outer pipe (d_ho_) can be estimated mathematically using Eq. (1) [4].
3. $d_{ho}=\frac{4 \times A}{P}= D_{o}{- D}_{i}$ (1)
4. Where A is the flow area (m^2^) and determined as ($\frac{\pi}{4} \times\left( D_{o}^{2}- D_{i}^{2} \right))$, $D_{i}$ is inner tube diameter, $D_{o}$ is outer shell diameter, and P is wetted perimeter.
5. Prandtl number (Pr) of the hybrid nanofluids' are figured out by the using Eq. (2) with the help of viscosity, specific heat, and thermal conductivity respectively [4].
6. ${Pr}_{hnf}= \left( \frac{\mu\times Cp}{k} \right)_{hnf}$ (2)
7. The heat transfer rates (Q) for hold and cold fluids, reveilles the heat gain of the cold fluid (Q_hybnf_) and the heat loss of the hot fluid (Q_h_) should be equal with reference to energy balance equation i.e. Q_hybnf_ = Q_hf_, from the current studies, an average of 4.21% deviation is observed, the rate of heat flow in tube side and annulus side are computed utilizing Eq. (3a) and (3b) [4].
8. $Q_{hybnf}= {Cp}_{hybnf}\timesṁ_{hybnf}\times{(T}_{hybnf,o}-T_{hybnf,i})$ (3a)
9. $Q_{hf}= ṁ_{h}\times{Cp}_{h}\times{(T}_{h,i}- T_{h,o})$ (3b)
10. Eq. (3c) provides the average rate of heat transfer in watts
11. $Q_{avg}= \frac{{(Q}_{hf}+ Q_{hybnf})}{2}$ (3c)
12. Where Q is Heat transfer rate, subscripts i and o stands for inlet and outlet respectively, ṁ mass flow rate of fluid, $T_{hybnf}$ temperature of cold fluid, Cp specific heat, and $T_{h}$ temperature of hot fluid respectively.
13. “Re” is used to indicate type of flow regime based on the fluid's flow rates, density, diameter, and viscosity. By utilizing Eq. (4a and 4b) “Re” is estimated hot stream as well as for cold stream [16].

${Re}_{hf}=\left( \frac{{\rho\times D}_{i} \times v}{\mu} \right) \left( 4a \right)$

14. ${Re}_{hybnf}=\left( \frac{{v\times D}_{o}\times\rho}{\mu} \right)$ (4b)
15. Where ${{Re}_{hf} and Re}_{hybnf}$ denotes Re for hot fluid and hybrid nanofluids respectively.
16. For counterflow pattern, the logarithmic mean temperature difference (LMTD) is computed by from Eq. (5) below [15]:
17. ${\Delta T}_{LMTD}= \frac{\left( T_{hf,i}- T_{c,o} \right)-\left( T_{hf,o}- T_{c,i} \right)}{ln\frac{\left( T_{hf,i} - T_{c,o} \right)}{\left( T_{hf,o}- T_{c,i} \right)}}$ (5)
18. Where $T_{hf,i}, {and T}_{hf,o}$ are temperatures of hot fluid at inlet and outlet and $T_{c,i}, T_{c,o}$ are temperature of cold fluid at inlet and outlet respectively.

Eq. (6) is used to calculate the average temperatures of hot fluid ($T_{hf avg})$ and cold fluid ($T_{c avg})$ [15]:

$T_{hf avg}= \frac{{(T}_{h,o}+ T_{h,i})}{2} {and T}_{c avg}= \frac{{(T}_{c,o}+ T_{c,i})}{2}$

2. (6)

The overall heat transfer coefficient (U) is computed by using Eq. (7) [18],

1. $U=\left( \frac{Q_{avg}}{LMTD \times A} \right)$ (7)
2. Where Q_avg_ average heat transfer rate (W), U is overall heat transfer coefficient (W/m^2^. k), and A area of heat transfer (m^2^).

The Sieder and Tate equation has been used to calculate the Nusselt number since the determined Reynolds number (Re) <2300, which is classified as laminar flow [56,57]. The following Nusselt number Eq. (8a) and (8b) were applied to the shell side and tube side of DPHE respectively.

1. ${Nu}_{o}=1.86\times\left( \frac{{P_{r}\times D_{o}\times Re}_{nf}}{L} \right)^{0.333}\times\left( \frac{\mu_{b}}{\mu_{w}} \right)^{0.14}$ (8a)
2. ${Nu}_{i}=1.86\times\left( \frac{{P_{r}\times D_{i} \times Re}_{i}}{L} \right)^{0.333}\times\left( \frac{\mu_{nf}}{\mu_{w}} \right)^{0.14}$ (8b)
3. $\mu_{w}$ is determined using the wall temperature and T_wall_ could be approximated from Eq. (9) [37,38]:
4. $T_{wall}\cong0.5\times\left( \left( \frac{T_{i}+T_{o}}{2} \right)+\left( \frac{t_{o}+t_{i}}{2} \right) \right)$ (9)
5. The hydraulic diameter (d_ho_), the Nusselt number (Nu), and the thermal conductivity values of hybrid nanofluids (k_hybnf_) have been applied for estimating the annulus heat transfer coefficient (h_o_), by using Eqs. (10) and (8a) respectively [18].
6. $h_{o}= \frac{{(N}_{uo}\times k_{hybnf})}{d_{ho}}$ (10)
7. Eq. (11) represents friction factor under laminar flow in tube side as well as shell side and computed by using the Darcy friction factor equation. For, flow through annulus i.e., outer pipe (D_o_), equation is given as [57]:
8. $f_{i}={\frac{64}{Re}}_{i}$ (11a)
9. $f_{o}={\frac{64}{Re}}_{nf}\times\left( \frac{{(1+k}^{2})}{\left( 1-k \right)^{2}}+ \frac{\left( 1+k \right)}{\left( 1-k \right) \times lnk} \right)^{-1}$ (11b)
10. Where k = $\frac{D_{i}}{D_{o}}$

Eq. (12) has been utilized to derive the pressure drop (∆P) in a heat exchanger

1. $f_{hf}=\frac{{\Delta P}_{hf}}{\left( \frac{L}{D_{i}} \right) \times\left( \frac{v^{2} \times\rho_{hf}}{2} \right)}$ (12a)
2. $f_{hybnf}=\frac{{\Delta P}_{hnf}}{\left( \frac{L}{D_{o}} \right) \times\left( \frac{v^{2}\times\rho_{hybnf}}{2} \right)}$ (12b)
3. Where $v$ is velocity of the fluid in (m/s)
4. The following Eq. (13) was used to evaluate the performance evaluation criteria (PEC) (or) thermal performance factor (TPF) defined by Webb et al. [58] by considering Nusselt number enhancement ratio to frictional losses [19,25,58-61].
5. $PEC=\frac{\left( \frac{{Nu}_{hybnf}}{{Nu}_{bf}} \right)}{\left( \frac{f_{hybnf}}{f_{bf}} \right)^{\left( \frac{1}{3} \right)}}$ (13)
6. Where ${Nu}_{hybnf}$, ${Nu}_{bf}$, $f_{hybnf}$ and $f_{bf}$ are Nusselt number and frictional losses for hybrid nanofluids and base fluids respectively.
7. Eq.14 is used to obtain percentage deviation while evaluating accuracy in terms of deviation [62].
8. $\% Deviation=\frac{\left( Predicted - Experimental \right)}{Experimental}\times100$ (14)
10. The following Eq. (15) was applied to figure out the percentage enhancement.
11. $\% e=\left( \frac{P_{hybnf}- P_{bf}}{P_{bf}} \right)\times100$ (15)
12. Where P stands for attributes such as the LMTD, Nu, h, *f*, ΔP, and U respectively. While $e$ stands for the percentage of enhancement.
13. The Shah correlation and Hausen correlation [66,67] represented in Eq. (16,17)
14. If ${{Re}_{nf}\times P}_{rnf}\times\left( \frac{D_{o}}{L} \right)\geq33.33, \mathrm{then}$
15. ${Nu}_{hynf}=1.953\times\left( \left( \left( \frac{D_{o}}{L} \right)\times Pr{\times Re}_{hynf} \right)^{0.3333} \right)$ (16)
16. If $0.1<P_{rnf}\times\left( \frac{d_{e}}{L} \right)\times{Re}_{hynf}< {10}^{4}$, then
17. ${Nu}_{hynf}=3.66+\left( \frac{\left( 0.19 \times\left( {{Re}_{nf}\times P}_{rnf}\times\left( \frac{D_{o}}{L} \right) \right)^{0.8} \right)}{\left( 1+0.117 \times\left( {\left( \frac{D_{o}}{L} \right)\times P}_{rnf}\times{Re}_{nf} \right)^{0.467} \right)} \right)$ (17)

Where ${Nu}_{nf}$ represents nanofluid Nusselt number, ${Pr}_{nf}$ shows nanofluid Prandtl number, ${Re}_{nf}$ signifies nanofluid Reynolds number, $D_{o}$ designates outer pipe diameter, and L represents length of heat exchanger.

1. Cost analysis can be determined by considering weighted average capital, equipment installation, service, and maintenance costs [69]. According to Hall method [70], cost analysis was carried out by considering the HEX standards, flow characteristics, and framework of material.
2. The heat exchanger's overall cost is determined using the Hall approach using Eq. (18).
3. $C_{O}= C_{\mathrm{YO}}+C_{\mathrm{UI}}$ (18)
4. Where C_O_, C_YO_, and C_UI_ are overall cost, yearly operational cost, and up-front investment cost.
5. The area of the heat exchanger affects the up-front investment cost, which may be estimated with the assistance equation (19).
6. $C_{\mathrm{UI}}=a_{1}+(A^{a3}\times a_{2})$ (19)
7. Where A is heat exchanger’s area (m^2^)
8. For stainless steel material, a_1_, a_2_, and a_3_ values were taken as 8000, 259.2, and 0.9 respectively [71].
9. The entire yearly operational cost is calculated using Eq. (20) by summing up the cost of pumping power to compensate for pressure losses.
10. $C_{\mathrm{YO}}=\sum_{K=1}^{n} \frac{C_{y}}{{(1+i)}^{K'}}$ (20)
11. Where C_y_ refers to annual operation cost and it is estimated by Eq. (21),
12. $C_{y}=H\times P\times C_{u}$ (21)
13. Where H and C_u_ stands for overall yearly operating hours and utility cost respectively.
14. $P= \frac{1}{Ƞ}\times\left( \left( \frac{ṁ_{t}}{\rho_{t}} \right)\times\Delta P_{t}+(\frac{ṁ_{s}}{\rho_{s}})\times\Delta P_{s} \right)$ (22)
15. Where ṁ, ΔP, ρ, and Ƞ represents mass flow rate (kg/s), pressure drop (Pa), density, and pump efficiency (70%)

Eq. (23) - (27) are used to figure out the uncertainties related to Re, Q, Nu, h, and *f* [76].

1. For heat transfer rate, uncertainty is calculated as below.
2. $u_{,Qc}=\sqrt{{\left( \frac{\Delta T}{T} \right)^{2}{+\left( \frac{\Deltaṁ_{c}}{ṁ_{c}} \right)}^{2}+\left( \frac{\Delta C_{\mathrm{pc}}}{C_{\mathrm{pc}}} \right)}^{2}}$ (23)
4. For Nusselt number, uncertainty is calculated as below.
5. $u_{,Nu}= \sqrt{\left( \frac{\Delta D_{h}}{D_{h}} \right)^{2}+\left( \frac{\Delta k}{k} \right)^{2}+\left( \frac{\Delta h}{h} \right)^{2}}$ (24)
6. For heat transfer coefficient (h), uncertainty is calculated as below.
7. $u_{,h}= \sqrt{\left( \frac{\Delta T}{T} \right)^{2}+\left( \frac{\Delta Q}{Q} \right)^{2}+\left( \frac{\Delta A}{A} \right)^{2}}$ (25)
8. For Reynolds number (Re), uncertainty is calculated as below.
9. $u_{,Re}= \sqrt{{\left( \frac{\Delta\rho}{\rho} \right)^{2}+\left( \frac{\Delta\mu}{\mu} \right)^{2}+\left( \frac{\Delta D_{h}}{D_{h}} \right)}^{2}+\left( \frac{\Delta v}{v} \right)^{2}}$ (26)
10. For friction factor (*f*), uncertainty is calculated as below.
11. $u_{,f}= \sqrt{{\left( \frac{\Delta L}{L} \right)^{2}+\left( \frac{\Delta D_{h}}{D_{h}} \right)}^{2}+\left( \frac{\Delta v}{v} \right)^{2}+\left( \frac{\Delta\rho}{\rho} \right)^{2}+\left( \frac{\Delta P}{P} \right)^{2}}$ (27)
